# Recent Progress of Soft and Bioactive Materials in Flexible Bioelectronics

**DOI:** 10.34133/cbsystems.0192

**Published:** 2025-04-29

**Authors:** Xiaojun Wu, Yuanming Ye, Mubai Sun, Yongfeng Mei, Bowen Ji, Ming Wang, Enming Song

**Affiliations:** ^1^ Institute of Optoelectronics & Department of Materials Science, Shanghai Frontiers Science Research Base of Intelligent Optoelectronics and Perception, State Key Laboratory of Integrated Chips and Systems (SKLICS), Fudan University, Shanghai 200438, China.; ^2^ State Key Laboratory of Medical Neurobiology and MOE Frontiers Center for Brain Science, State Key Laboratory of Molecular Engineering of Polymer, Fudan University, Shanghai 200438, China.; ^3^ Unmanned System Research Institute, National Key Laboratory of Unmanned Aerial Vehicle Technology, Integrated Research and Development Platform of Unmanned Aerial Vehicle Technology, Northwestern Polytechnical University, Xi’an 710072, China.; ^4^ Queen Mary University of London Engineering School, Northwestern Polytechnical University, Xi’an 710072, China.; ^5^ Institute of Agro-food Technology, Jilin Academy of Agricultural Sciences (Northeast Agricultural Research Center of China), Changchun, China.; ^6^ International Institute for Intelligent Nanorobots and Nanosystems, Neuromodulation and Brain-machine-interface Centre, Fudan University, Shanghai 200438, China.; ^7^ Yiwu Research Institute of Fudan University, Yiwu, Zhejiang 322000, China.; ^8^ Frontier Institute of Chip and System, Fudan University, Shanghai 200433, China.

## Abstract

Materials that establish functional, stable interfaces to targeted tissues for long-term monitoring/stimulation equipped with diagnostic/therapeutic capabilities represent breakthroughs in biomedical research and clinical medicine. A fundamental challenge is the mechanical and chemical mismatch between tissues and implants that ultimately results in device failure for corrosion by biofluids and associated foreign body response. Of particular interest is in the development of bioactive materials at the level of chemistry and mechanics for high-performance, minimally invasive function, simultaneously with tissue-like compliance and in vivo biocompatibility. This review summarizes the most recent progress for these purposes, with an emphasis on material properties such as foreign body response, on integration schemes with biological tissues, and on their use as bioelectronic platforms. The article begins with an overview of emerging classes of material platforms for bio-integration with proven utility in live animal models, as high performance and stable interfaces with different form factors. Subsequent sections review various classes of flexible, soft tissue-like materials, ranging from self-healing hydrogel/elastomer to bio-adhesive composites and to bioactive materials. Additional discussions highlight examples of active bioelectronic systems that support electrophysiological mapping, stimulation, and drug delivery as treatments of related diseases, at spatiotemporal resolutions that span from the cellular level to organ-scale dimension. Envisioned applications involve advanced implants for brain, cardiac, and other organ systems, with capabilities of bioactive materials that offer stability for human subjects and live animal models. Results will inspire continuing advancements in functions and benign interfaces to biological systems, thus yielding therapy and diagnostics for human healthcare.

## Introduction

Flexible bioelectronics can bridge the gap between mechanical devices and living organisms and play critical roles in real-time monitoring, sensing, recognizing, and actuating [1–5]. However, the mechanical mismatch between electrodes and soft tissue hinders their conformable usage. For example, the Young’s modulus of myocardial tissue ranges from 10 to 15 kPa, while that of electronic materials is up to 100 GPa [6]. The state-of-the-art electrocardiogram (ECG) signal acquisition relies heavily on in vitro wearable devices, e.g., Holter devices, whose accuracy feedback is impacted by dynamic movement and transdermal transport manner [7,8]. Next-generation bioelectronics is characterized with advantages including soft, ultrathin, light weight, and microscale, which can be used for implantable application to avoid those drawbacks [9,10]. Meanwhile, they can contribute to the development of precise and personalized medicine by supporting continuous long-term monitoring [11,12].

Material engineering and structure engineering, which focus on silicon thin film and its configuration design, are the crucial foundation of next-generation bioelectronics. The wide operation temperature range (*T*_m_ ~ 1,420 °C) of monocrystalline silicon allows it to undergo various doping and manufacturing processes that enable its application in the integrated semiconductor device [13]. Since bulk silicon is rigid and high energy consuming, silicon-based thin-film membranes with a nanometer scale structural dimensions emerge to meet the soft and conformal requirements [11,14–16]. In addition to the thickness reduction of silicon substrates, their configuration can be fabricated into open-mesh/honeycomb porous structures, serpentine patterns, and kirigami/origami structures with intrinsic stretchability, further benefiting the mechanical match of bioelectronics and living organisms [2,10,17–21]. Other novel materials have shown their competitiveness in intrinsically stretchable bioelectronics design, e.g., rubber, polyurethane (PU), and hydrogel scaffolds [22–25]. Recently, advanced manufacturing technologies also contribute to the flourishing development of bioelectronics, e.g., 3-dimensional (3D) printing and microfluidic technologies, which endow bioelectronics with sophisticated module design in both components and structures and can be integrated with therapeutic capabilities [26–28].

Silicon can offer the in situ growth of insulation layer SiO_2_ by thermal oxidation, which is water-insoluble and possesses excellent chemical stability and insulation properties [29]. In general, bioelectronics encapsulated by SiO_2_ thin film would work stably in practical applications, while the local environment change over implanting makes it vulnerable. The interface of implanted devices is first attacked by foreign body response (FBR), followed by congregation of proteins, neutrophils, fibroblasts, macrophages, and so on [20,30]. Under the electric field, the encapsulation layer becomes sensitive to water and ions, accelerating the failure of devices. With prolonged implantation, fibrous capsule formation and inflammation would decrease the monitoring reliability and lead to device failure eventually [31,32]. Many efforts have been devoted into guaranteeing the chronic implantable signal acquisition of bioelectronic from several aspects: (a) mechanical match is necessary to minimize the mechanical damage of target tissue during implanting and enhancing the conformable contact; (b) the chemical interface is biocompatible or immune shielding to avoid FBR; (c) anti-inflammatory or immunosuppressant is adopted to modulate an immune response. Besides chronic application, some bioelectronics are designed with short-term service, where programmable biodegradability is highly demanding [33–35]. In both cases, the degradation products should be bioabsorbable or metabolized by the host.

Nowadays, bioelectronics using multifunctionalization (BUMF) is blooming, and based on this, the integration of diagnosis (monitoring) and therapy (stimulation) has emerged [36–38]. For example, hydrogels have been applied as a tensile encapsulation material for bioelectronics, and they can also be used as drug carriers with sustained release [39–42]. Considering that novel active materials can benefit bioelectronic innovations (e.g., intrinsic stretchable, chronic implantable, and programmable biodegradable) and play a vital role in the construction of BUMF, we would give a systemic review of the materials design and development.

In this review, the development and application of implantable bioelectronics over the past few years is reviewed. We highlight the promising material design that contributes to bioelectronics progress from the viewpoint of mechanical and biological. Specially, soft, flexible, and self-healing materials that benefit reliability of bioelectronics in dynamic environment from the aspect of mechanical match are introduced systemically. Bioadhesive and bioactive materials that favor the robust, biosafe, and multifunctional bioelectronics from the aspect of biological research are also summarized. Applications of implantable bioelectronics in monitoring and sensing, modulation of physiological and pathological conditions via smart response, and tissue regeneration via electrical stimulation are summarized later. Furthermore, we discuss the remaining challenges and opportunities for application of bioelectronics in precise diagnosis and treatment. We expect to provide comprehensive references for researchers who are seeking a roadmap toward the de novo design of bioelectronics for in vivo application.

## Flexible and Implantable Bioelectronics

**Fig. 1.**
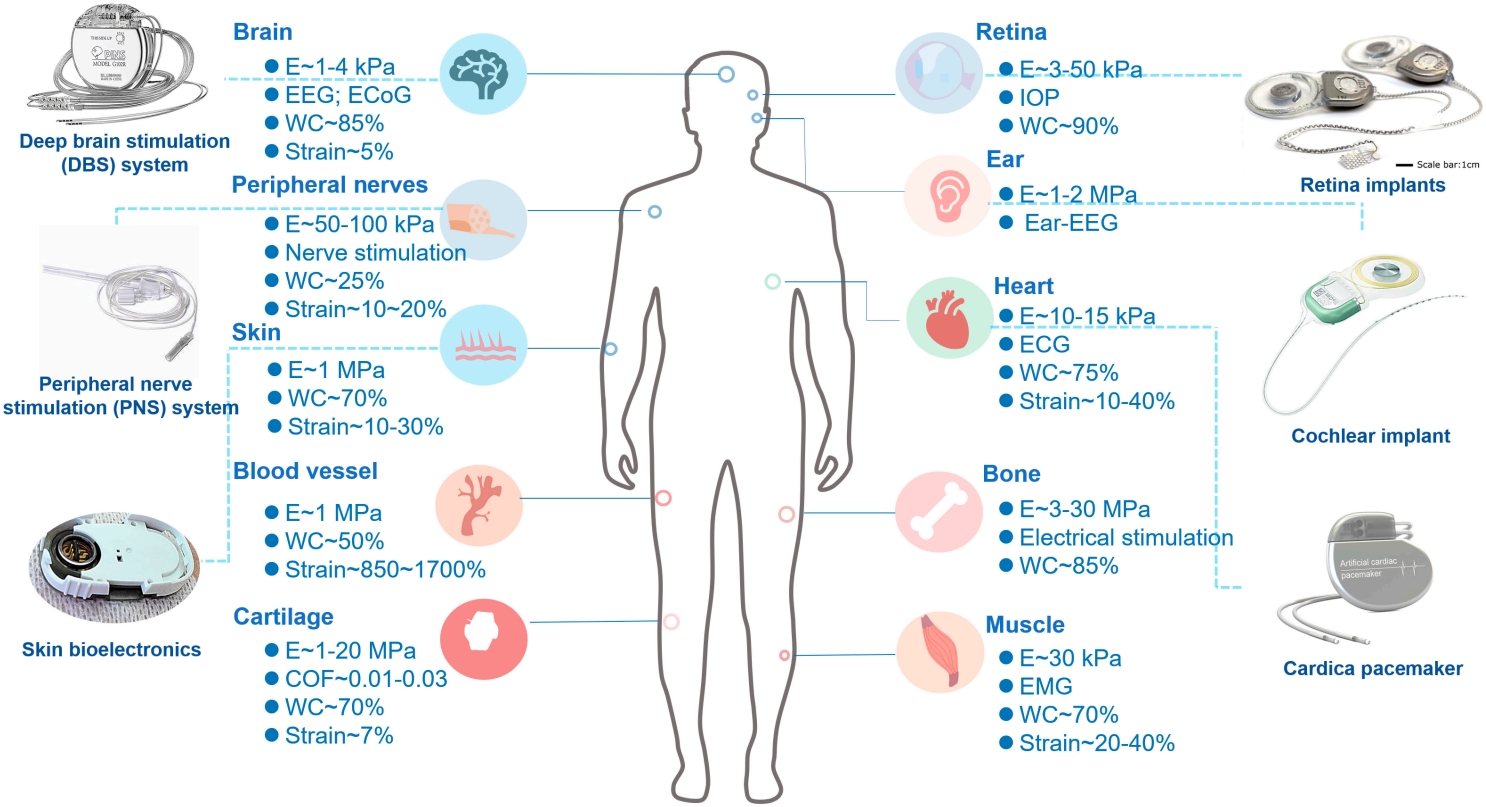
Typical applications of implantable bioelectronics for healthcare toward organs/tissues with different biomechanical feature. *E*, elastic modulus; EEG, electroencephalography; ECoG, electrocorticography; WC, water content; COF, coefficient of friction; IOP, intraocular pressure; ECG, electrocardiogram; EMG, electromyography.

Currently, in vivo implantation of bioelectronics has gained great attention due to the high signal-to-noise ratio (SNR) and precise information feedback on target tissues. Fig. 1 demonstrates typical applications of implantable bioelectronics toward different organs/tissues for healthcare. Organs and tissues, including brain, heart, muscle, neural, and retina, possess different biomechanical features, giving distinct requirements for bioelectronics [6,43–45]. For their different implantable purposes, chronic and transient bioelectronics need distinct encapsulation strategies. However, flexible bioelectronics is the main development tendency due to its advantages in mechanical match and conformal intact with soft tissues [2,8,46,47]. In this section, based on the latest progress in soft, flexible, stretchable bioelectronic devices, we discuss the general principle recognized in their design and limitations in practical application.

### Design principle of flexible and stretchable bioelectronics

Metals and their oxides or nitrides, such as platinum, gold, iridium, and iridium oxide, are commonly employed to give a low impedance and biocompatibility interface with tissue and bioelectronics [48]. Utah array and Michigan probes represent the 2 mature implants for recording signals from the brain. In particular, Utah array becomes the “gold standard” for high-channel brain–computer interface (BCI) and has been implanted in rats, monkeys, and even patients [48–51]. Yet, the rigid probes might fail for the dynamic nature and FBR of tissues over chronic implantation [52]. Considering the local environment of bioelectronics, strategies including surface modification, soft encapsulation, and bio-glue have been adopted to prolong the lifetime of bioelectronics.

**Fig. 2.**
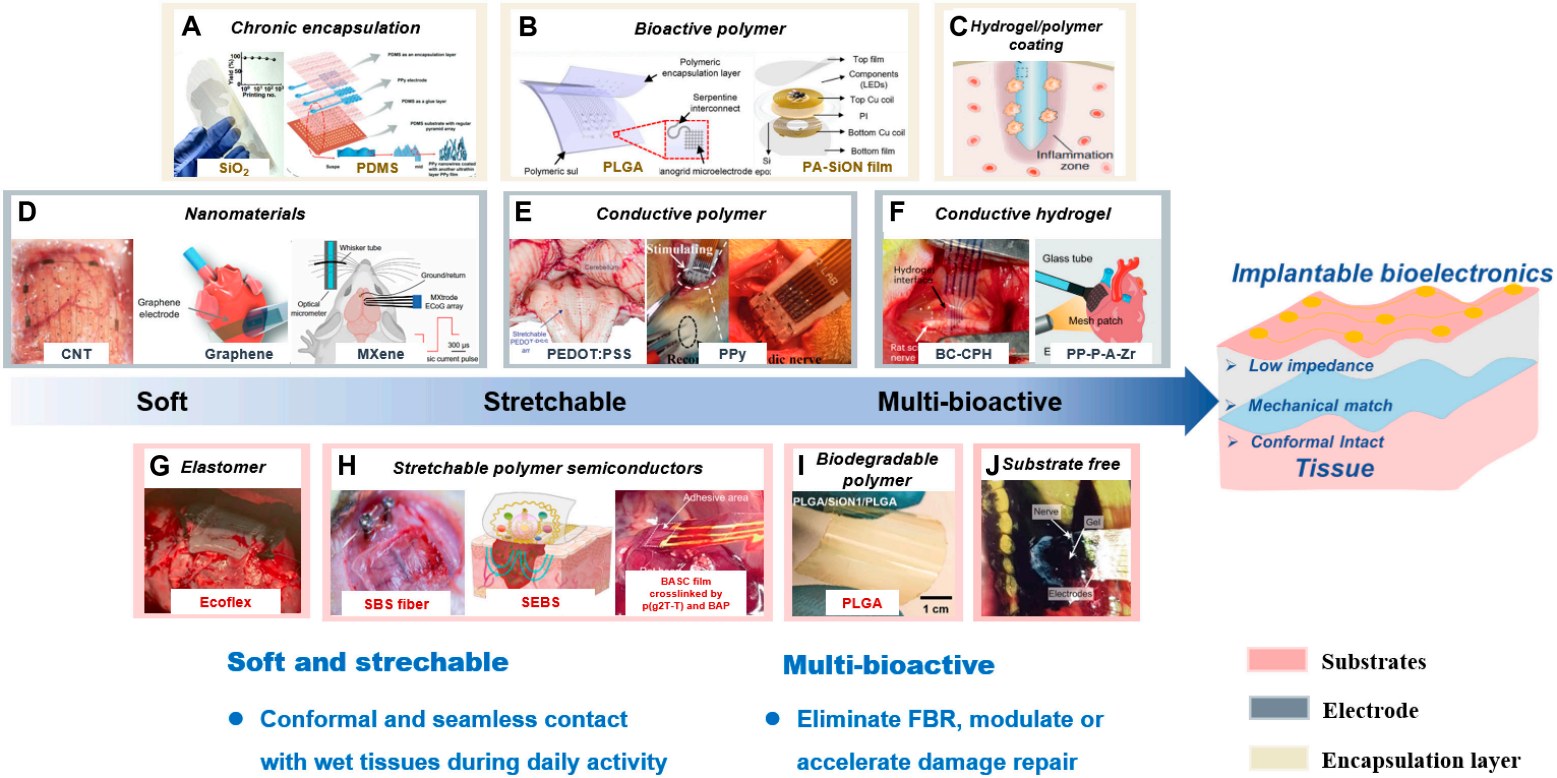
Brief overview of recent development of implantable bioelectronics. (A to C) Encapsulation layers. (A) Chronic encapsulation: SiO_2_. PDMS: Reproduced with permission from [42]. Copyright 2023, Wiley-VCH. (B) Bioactive polymer for biodegradable encapsulation: PLGA: Reproduced with permission from [72]. Copyright 2024, Wiley-VCH. PA-SiON: Reproduced with permission from [73]. Copyright 2024, Wiley-VCH. (C) Hydrogel encapsulation: Hydrogel/polymer coating: Reproduced with permission from [74]. Copyright 2023, Wiley-VCH. (D to F) Electrode. (D) Conductive nanomaterials as electrode: CNT: Reproduced with permission from [244]. Copyright 2024, Springer Nature Limited. Graphene: Reproduced with permission from [53]. Copyright 2023, Wiley-VCH. MXene: Reproduced with permission from [54]. (E) Conductive polymer as soft electrode: PEDOT:PSS: Reproduced with permission from [55]. Copyright 2022, AAAS. PPy: Reproduced with permission from [56]. Copyright 2017, Wiley-VCH. (F) Conductive hydrogel as soft electrode: BC-CPH: Reproduced with permission from [26]. Copyright 2023, Springer Nature Limited. PP-P-A-Zr: Reproduced with permission from [245]. Copyright 2024, Wiley-VCH. (G to J) Substrate. (G) Silicon-based elastomer: Ecoflex: Reproduced with permission from [76]. Copyright 2024, Elsevier. (H) Stretchable polymer semiconductors: SBS fiber: Reproduced with permission from [77]. Copyright 2023, AAAS. Self-adhesive SBES: Reproduced with permission from [75]. Copyright 2023, AAAS. BASC film crosslinked by p(g2T-T) and BAP: Reproduced with permission from [78]. Copyright 2023, AAAS. (I) Biodegradable polymer: PLGA: Reproduced with permission from [79]. Copyright 2024, Wiley-VCH. (J) Substrate free: Reproduced with permission from [80]. Copyright 2023, AAAS.

As shown in Fig. 2, we systemically summarize the recent development of bioelectronics from viewpoints of electrode, encapsulation layer, and substrates. Novel materials can boost remarkably the soft and stretchable performance of bioelectronics. Carbon-based materials [e.g., graphene, MXenes, and carbon nanotubes (CNTs)] and conducting polymers (CPs) [e.g., poly (3-4, ethylenedioxythiophene) (PEDOT), polypyrrole (PPy), and polyaniline (PNAi)] have been applied to construct flexible and stretchable bioelectronics [36,53–63]. Moreover, conductive polymer, hydrogel, semi-dry hydrogel, or elastomers are developed and employed as replacements or coatings of rigid electrodes for improved mechanical match and conformability with tissues [18,41,56,64–71]. Insulators and encapsulation layer mainly determine the lifetime and mechanical performance of bioelectronics. In addition to SiO_2_, soft polymers such as polydimethylsiloxane (PDMS), Ecoflex, polyimide (PI), and parylene C are used for chronic flexible thin-film encapsulation, while polycaprolactone (PCL), polylactic-glycolic acid (PLGA), and polyglycolic (PGA) are applied to biodegradable and bioabsorbable bioelectronic encapsulation [42,72–75]. Substrates for bioelectronics are limited by the processing conditions; hence, rubbery, PU, and stretchable polymer semiconductors with intrinsic stretchable and self-healing properties are also proposed [75–81]. In addition, wireless data transmission and energy supply are also in hot research for implantable bioelectronics, although they are not covered in this review [2,20]. These efforts contribute to the development of implantable bioelectronics with good biocompatibility, designable lifetime, high signal quality, and high reliability.

Although material systems with unique feature can meet the requirement of application of bioelectronics toward a specific condition, their composition shall be adjustable considering the overall performance about mechanical, electrical, biochemical, and so on. Mechanical properties such as tensile strength, Young’s modulus, and fracture toughness shall in similar level with “soft and wet” tissues to eliminate mechanical mismatch. Besides high electrical conductivity for signal sensing and amplification, the circuit can provide additional modulation via external stimulus such as force, photo, acoustic, and magnetic. Biochemical properties, especially the surface biochemistry, account for the direct interaction with tissues and can also provide surface sensing and shielding function. Nowadays, all-soft bioelectronics that are promising in connect tissue engineering and bioelectronics may provide a platform for seamless HMI. We inferred it as the main tendency for bioelectronics.

### Design and fabrication of flexible and stretchable bioelectronics

#### Soft and stretchable materials

Rigid and thick implants cause more serious FBR and mechanical mismatch than the soft and thin ones; thus, implantable bioelectronics are moving toward thinner and flexible implants. Silicon-on-insulator (SOI) technologies bring a thin platform for the fabrication of the hyperflexible device [33,82]. With the development of nearly 40 years, SOI wafers can be polished with a thickness of less than 100 nm. Devices based on those wafers can stand deformation such as bending, twisting, and folding [83]. For instance, ultrathin neuroelectronic array based on n-doping of Si nanomembrane with a thickness of ~140 nm demonstrated negligible performance degradation after 10,000 bending cycles (bend radius ~ 4 mm) [4]. With the aid of transfer printing, devices can be integrated on soft substrate, e.g., PDMS, PI, and other polymer films [cellulose, silk fiber, poly(vinyl alcohol) (PVA), and so on] [84–87]. Eye-implantable probe (width: 190 μm, thickness: 110 μm) was fabricated via transfer printing by PDMS stamp to PI panel with SU-8 as adhesive layer [88]. The flexible, small size, and probe-type pressure-sensitive transistor can selectively and preciously monitor intraocular pressure (IOP), without the risk of eye damage and measurement errors.

Combined with structural design such as waves, helices, open-mesh serpentine, and island-bridge structures, the resulting flexible 3D structure can afford stretch deformation [11]. Brainmask with fractal serpentine design and bacterial cellulose (BC) substrate was reported [87]. The Brainmask device kept the structure intact after attaching to the agar gel cylinder (5 mm in radius). Meanwhile, high moisture of BC film makes it easily attach to the nonplanar epidural surface that favors the precise recording of in vivo electrocorticography (ECoG) signals. 3D structures that show more accommodation to curved surface of organ/tissues can also be the target of transfer printing. Combined with structural design such as waves, helices, open-mesh serpentine, and island-bridge structures, the resulting flexible 3D structure could afford stretch deformation. Yu and colleagues [89] report a biaxially stretchable curvy, shape-adaptive imager with balloon stamp transfer, and kirigami structural design. Under 30% biaxial strain, the optoelectronic 32 × 32-pixel array can maintain 78% of its electrical performance.

Elastomers such as PU, rubbery, and stretchable polymer semiconductors [e.g., poly-thieno[3,2-b]thiophene-diketopyrrolopyrrole (DPPTT)] are promoted with intrinsically high elasticity and low hysteresis [89]. Deformable bioelectronics based on those elastomers can be applied for conditions with large deformations, like beating heart. Epicardial bioelectronic patch based on rubbery presents suitable mechanical softness to heart tissue that can realize spatiotemporal mapping of electrophysiological activity [18]. Hydrophilic PU composited with conductive fillers [e.g., CNT, poly(3,4-ethylenedioxythiophene)-polystyrene sulfonate (PEDOT:PSS), and liquid metal] can be engineered to be multifunctional bioelectronics [90,91]. A tissue adhesive ink consisting of PU, poly(acrylic acid) N-hydroxy succinimide ester (PAA-NHS), and ethanol/water mixture was fabricated into tissue adhesive patches with conformable, stretchable, and robust adhesion with wet tissues within seconds [92]. Conductive silver ink was employed to construct a simple LED circuit to explore the bioadhesive bioelectronic patch application of the 3D printing platform. Meanwhile, modification of elastomers to improve their reliability is ongoing. Very recently, a hydrophilic polyurethane (RPU) substrate was reported with high binding strength to wet gold (Au) grains (1,243.4 N/m), which is superior to pervious elastomeric polymers [PDMS, styreneethylene-butylene-styrene (SEBS), and Ecoflex] [86].

Hydrogel is another representative material for soft and stretchable bioelectronics, and has shown outstanding performance for its extracellular matrix (ECM)-like structure and composition. Hydrogel-based cardiac patch can not only provide real-time monitoring of myocardium electrical activity but also modulate tissue damage via drug delivery or electronic stimulation [60,93,94]. Despite promising mechanical strength of hydrogel-based bioelectronics, the chronic implantation of hydrogel is difficult as it is prone to the attack or corrosion by surrounding body fluid.

#### Self-healing materials

Self-healing materials rapidly and spontaneously restore their structures and functionalities upon encountering damage under external stimulations, which can enhance the anti-fatigue performance of devices, extend their lifespans, and improve their reliability [95–97]. These materials offer numerous advantages for minimally invasive surgeries aiming at tissue regeneration, including nerve regeneration, wound healing, implantable prosthetics, bone repair, and tendon repair [62,98–101]. Restoration of the original structure, mechanical properties, and electrical conductivity is achieved through various mechanisms, such as noncovalent bonds (e.g., electrostatic interactions, metal coordination interactions, and hydrogen bonding interactions) or dynamic reversible covalent chemical bonds (such as Diels–Alder reactions and Schiff base reactions) [95]. Materials capable of spontaneous and rapid repair in the in vivo environment are particularly suited for practical applications. Implantable self-healing materials are primarily categorized into hydrogels, elastomers, and conjugated polymers.

**Fig. 3.**
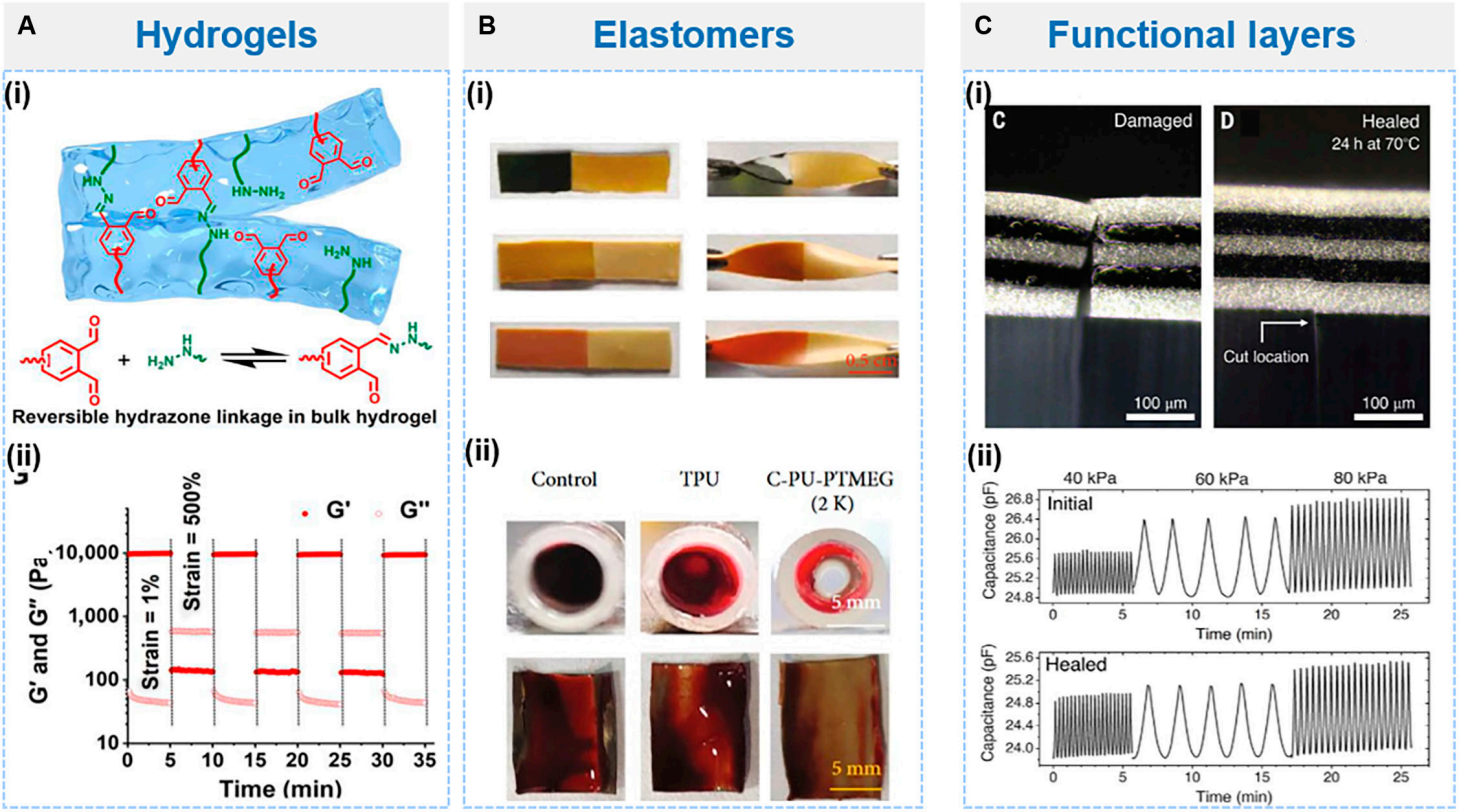
Self-healing hydrogels and elastomers. (A) Self-healing hydrogels. (i) Dynamic cross-linking in the hydrogel between HA and 4ApeG-OPA. (ii) Step–strain test for 7% (w/v) HA-PEG hydrogel. Reproduced with permission from [103]. (B) Self-healing elastomers. (i) The self-healing of the family of C-PU-PTMEG elastomers in PBS. (ii) Less thrombus coverage for the C-PU-PTMEG-treated group at the cross-section of the catheter. Reproduced with permission from [108]. Copyright 2022, AAAS. (C) (i) Fracture capacitor (left) and healed capacitor with layer realignment (right). (ii) Initial (top) and healed (bottom) pressure-sensing performance of the capacitor. Reproduced with permission from [110]. Copyright 2023, AAAS.

Hydrogels have gained extensive attention owing to their tissue-like properties, including a similar Young’s modulus and compatibility with physiological microenvironments and tissues, which promote favorable biocompatibility and molecular transport [38,102]. Particularly, injectable self-healing hydrogels have garnered considerable attention due to their effectiveness in filling irregular defects. The basis for the self-repairing properties of hydrogels is the reversibility of the crosslinked network bonds. Self-healing hydrogels can be prepared by functionalizing conventional polymers, such as the natural polymer protein, cellulose, sodium alginate, chitosan, gelatin, and hyaluronic acid (HA), as well as the synthetic polymers including polyethylene glycol, polyetherimide, and polyethylene oxide. For example, Ren et al. [103] polymerized hydrazide-modified HA and o-phthalaldehyde (OPA)-terminated 4-armed poly(ethylene glycol) (4ApeG-OPA) into an injectable, self-healing hydrogel, which showed better wound healing efficiency than commercial glues (Fig. 3Ai). When a strain of 500% was applied to the 7% (w/v) HA-PEG hydrogel, both storage modulus (*G*′) and loss modulus (*G*″) values were fully recovered after the strain was reduced to 1% at 5-min time intervals (Fig. 3Aii). Moreover, Xu et al. [104] introduced a bioactive hydrogel featuring Schiff base bonding between tannic acid-modified gold nanocrosslinkers and chitosan, which rapidly recovered within 30 min. Injection of the hydrogel into the brains of Parkinson’s disease (PD) rats significantly mitigated irregular neuronal cell discharges in the subthalamic nucleus, thereby aiding in the restoration of motor function.

However, self-healing hydrogels relying on weak dynamic bonds often suffer from low toughness and susceptibility to in vivo breakage [99]. Elastomers represent another common class of self-healing materials characterized by adaptive mechanical properties and exceptional resilience. PDMS- and PU-based self-healing elastomers are prevalent in implantable devices, leveraging segment mobility, reversible covalent bonds, and hydrogen bonds to achieve self-healing capabilities [105–107]. The first stage for the self-healing of devices is the self-healing of elastomer substrates. Zhou et al. [108] developed catechol-functionalized polyurethane (C-PU-PTMEG) elastomers. By adjusting the molecular weight of poly(tetramethylene ether glycol) (PTMEG), they modulated the mobility of chain segments and dynamic hydrogen bonding to enhance material self-healing. At 37 °C, C-PU-PTMEG exhibited self-healing properties in air and phosphate-buffered saline (PBS) solution (Fig. 3Bi). When used as a cardiovascular scaffold coating on the inner wall of a commercial trigeminal vessel after connecting arteriovenous vessels in rabbits, it showed improved patency with reduced thrombus formation compared to commercial thermoplastic polyurethane (TPU) (Fig. 3Bii). Besides the elastomer substrate, the self-healing of conductive component is equally important in bioelectronic applications. Son et al. [109] embedded CNTs within a self-healing PDMS–MPU_0.4_–IU_0.6_ elastomer. The fractured conductive network autonomously regenerated, demonstrating complete restoration of the resistance–strain behavior after 12 h of self-healing, and capable of withstanding 100% tensile strain. Moreover, achieving self-healing of complex device shapes and functionalities also necessitates self-healing behavior between different functional layers. Cooper et al. [110] achieved automatic reorganization and healing between layers by alternately arranging PDMS-based polymer and polypropylene glycol (PPG)-based polymer multilayer films (Fig. 3Ci), utilizing post-damage surface energy differences. When applied in capacitive pressure sensors, the drifted and hysteresis changes in device capacitance after self-repair were minimal (Fig. 3Cii), with 96% recovery of initial capacitance after heating and the full recovery of mechanical and conductive properties after 72 h of annealing at 70 °C. Such interlayer co-self-healing characteristics prevent misalignment from constraining device functionality restoration. However, its self-healing capacity remains limited at room temperature.

Lastly, the self-healing properties of conjugated polymers have been studied to promote the development of devices based on organic semiconductors, including organic thin-film field-effect transistors. Oh et al. [111] designed a 3,6-di(thiophen-2-yl)-2,5-dihydropyrrolo[3,4-c]pyrrole-1,4-dione (DPP)-based conjugated polymer containing 2,6-pyridine dicarboxamide (PDCA). Through thermal annealing and solvent vapor annealing treatments, the mobility of damaged polymer films recovered from 0.024 to 1.13 cm^2^/Vs.

#### Tissue-like performance: Mechanical and functional properties

Tissue-like materials have been developed to create seamless and adaptive interface between electronic devices and human tissues, aiming to minimize mismatch [112]. Extensive research has focused on reducing the mechanical mismatch between implanted materials and surrounding tissues to mitigate immune responses, implant rejection, and foreign body reactions, thus enhancing biocompatibility [65,113,114]. Parameters such as hardness, tensile strength, toughness, viscoelasticity, relaxation time scale, adhesion, and solute diffusivity are crucial for characterizing mechanical suitability, among which Young’s modulus is of primary interest [115]. Recent material advancements have enabled implants to not only possess Young’s modulus similar to that of tissues but also adjust their mechanical properties as needed by employing strategies such as intrinsic softness and the design of soft interlayer structures [56].

Additionally, many tissues in the human body, including muscles, tendons, and articular cartilage, exhibit anisotropic structural characteristics [116,117]. Developing materials capable of effectively mimicking such anisotropic mechanical properties is crucial for preserving, replacing, and repairing these biological tissues. Recent strategies to achieve mechanical anisotropy involve stretching nanofibers, combining stretching and compression, waving, supramolecular assembly, and stretching polymer chains [118–122]. Additionally, bio-3D printing has emerged as a promising approach for fabricating anisotropic structures [121]. Furthermore, materials with both mechanical property and electrical conductivity similar to those of tissues have been developed. In particular, electroactive materials with high strength, which have been made into artificial muscles or artificial tendons, have received much attention. Artificial muscles have achieved a wide range of applications in mimicking muscle contraction to generate force, mechanical actuating robotics, and prosthetic limb. For instance, Pan et al. [122] proposed a supertough spider silk composites for artificial tendon, with a toughness of 420 MJ/m^3^ and a conductivity of 1,077 S/cm. The electro-tendon, which is composed of spider silk, single-walled carbon nanotubes (SWCNTs), and PEDOT:PSS, showed no change in conductivity after more than 40,000 bending and stretching cycles.

Moreover, in terms of functionality, materials mimicking tissue properties, such as drug delivery, self-healing, and stimulus-responsive materials, have been developed [40,97,123]. Conductive hydrogels, which have high softness, tissue-like mechanical properties, high water content, mimicry of the extracellular environment, and potential for multifunctionality, are emerging as promising candidates for tissue-like materials [112]. Park et al. [123] introduced an injectable, degradability-tunable conductive hydrogel. This hydrogel combines poly(ethylene glycol)-tetrathiol and thiol-functionalized reduced graphene oxide (rGO) with hydrolyzable poly(ethylene glycol)-diacrylate and poly(ethylene glycol)-dimaleimide, respectively. The resulting hydrogels exhibit a Young’s modulus of 15 to 17 kPa and an electrical conductivity of 21 to 22 mS/cm. Hydrolyzable conductive hydrogels vanish within 3 d after in vivo injection, while bioelectrodes based on stabilized conductive hydrogels can record electromyographic signals in vivo for up to 21 d, achieving an SNR of 7.03 ± 1.7.

Biocompatibility of the material with tissues also requires consideration for minimizing inflammatory or immune response to host tissue. Natural biomaterials including natural polysaccharide (such as chitosan, cellulose, starch, alginate, sodium hyaluronate, and dextran) and protein (such as silk, collagen, elastin, reflectin, and keratin) benefit the progress of stable bioelectronic interfaces [124,125]. Additionally, the structure and composition of bioactive materials are similar to the natural ECM, which facilitates cell proliferation and differentiation, and has great advantages in promoting tissue repair and wound healing. Wang et al. [126] designed bilayer hydrogels based on NHS ester-modified sodium alginate and chitosan. The hydrogel could spontaneously curl into microtubules from its initial film form to form an adaptive and conformal bioelectronic interface. Cells on its surface have very high cellular activity and proliferation ability, and are also able to form adhesion spots and further fully form intercellular linkages.

### Implantable bioelectronics systems

#### Foreign body response

The FBR governed by immune system can impend the lifetime and quality of implantable bioelectronics [31]. FBR is a process that can be divided into 3 stages: protein adsorption, matrix deposition, and fibrous encapsulation. Proteins from surrounding body fluids can contact and adsorb onto implants within several seconds and trigger an immune response. Protein aggregation is related to the surface property such as surface wettability, topography, and roughness, driving numerous works that focus on surface modification [30]. The superhydrophobic, superhydrophilic, and superwetting surface has been reported to resist the nonspecific binding of proteins and thus escape the subsequent immunorecognition [127,128]. Materials including PEG, zwitterionic, polypeptides, and polysaccharides have been employed in surface modification in the form of coating, hydrogel, membrane, polymer brush, and so on [129–131]. Drugs with anti-inflammation or immunosuppressive activity can also modulate FBR at implanted regions [132]. However, FBR is a continuous process where matrix deposition and fibrous encapsulation occur after blood protein adsorb, loading anti-inflammation drugs cannot prevent fibrous capsule formation eventually.

Matrix deposition is the result of protein adsorption, which consisted of acute and chronic inflammation [133]. Acute inflammation exists at the early stage of implantation (<7 d), during which polymorphonuclear neutrophils (PMNs) recruited by aggregated proteins are the primary cell types. PMNs would secrete proteolytic enzymes and reactive oxygen species (ROS) to defend against microorganisms, as well as chemoattractant [e.g., interleukin-8 (IL-8)] to appeal monocytes and macrophages migrated and polarized into pro-inflammatory M1 macrophages [134]. Chronic inflammation starts when monocytes and macrophages become predominant and fade over time (~21 d) [135]. During this period, M1 macrophages release ROS continuously and adhere and spread onto the implant surface to “engulf” it. Therefore, the implant device is wrapped with infused macrophages, which are called multinucleated foreign body giant cells (FBGCs) [136]. FBGCs work together with other immune cells to attract fibroblasts by secreting pro-fibrotic factors [including transforming growth factor-β (TGF-β), IL-6, platelet-derived growth factor (PDGF), and connective tissue growth factor (CTGF)]. Fibroblasts infiltrate and proliferate gradually, and deposit collagen to reconstruct the surroundings of devices, and capsules are formed by taking fibroblasts as major cells [137]. A stiffer and thicker capsule can be sensed and causes pain of adjacent tissues, even requiring secondary surgery [138,139]. Considering the cascade nature of FBR, materials with inherent histocompatibility, immunosuppression, and self-cleaning capability play a prominent role in the development of implantable bioelectronics. We would discuss the recent advances in facilitating HMI from the perspective of biomedical materials.

#### Tissue adhesive materials and architectures

Tissue adhesion ensures a seamless bioelectronics–tissue interaction, which favors the reliability and validity of bioelectronics. Generally, strategies to construct tissue adhesive interface include surface chemistry and topography modification. Mussel-inspired chemistry provides an efficient tool for preparation of wet tissue adhesive materials [140,141]. Dopamine, a typical catechol-based derivative, has been widely employed in the form of dopamine-grafted polymers or self-assembly polydopamine nanoparticles (PDA NPs) to supply material-independent adhesion [142,143]. Lu and colleagues [144,145] have focused their researches on the adhesive hydrogel with tough, self-healing, conductive, antifreezing, antioxidizing properties to monitor physiological signals and treat diseases. Considering the important role in implantable bioelectronics, they designed an ultrasoft, bioadhesive, transparent, conductive, and immune evasive hydrogel as brain–machine interface (BMI) by incorporating dopamine methacrylate-hybridized poly(3,4-ethylenedioxythiophene) nanoparticle (dPEDOT NP) into the carrageenan (CA) interpenetrated PDA-polyacrylamide (CA-PDA-PAM) network [145]. The hydrogel realized a conformal contact with brain tissue for its low modulus (<1 kPa) and high adhesion strength (~10 kPa). The catechol groups on the hydrogel help to integrate electronic device and soft brain tissue for both scalp electroencephalogram (EEG) and intracranial ECoG signal recording, with an SNR of ~10.19 dB for the ECoG signal. With the development of the wet adhesion mechanism, hydrophobic interaction is emphasized. They report a short alkyl chain-modified hydrogel in addition to dopamine. The PDA-PAM-C2 hydrogel composed of acrylamide (AM), dopamine, and ethyl acrylate (C2) possessed the highest adhesive strength to wet glass surfaces (23.5 ± 1.9 kPa) and reproducible adhesive strength to porcine skin (~8 kPa) [146]. Molecular dynamics (MD) simulation revealed that the hydration layer broken by C2 strengthened the interaction between the hydrogel and glass surfaces. A conductive hydrogel was fabricated by introducing a PDA-functionalized CNT (pCNT) for in vivo epicardial ECG signal detection, showing a potential for in vivo physiological signal monitoring and stimulation.

**Fig. 4.**
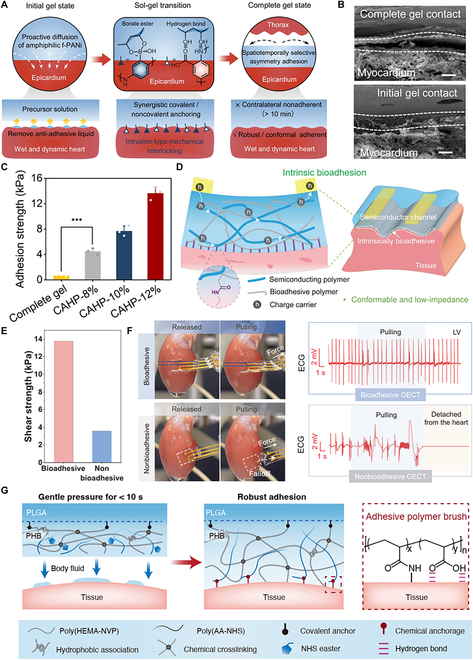
Wet tissue adhesive materials. (A) Chronological adhesion mechanism of the CAHP. (B) Scanning electron microscopy images of the hydrogel–tissue interface. (C) Adhesion strength between CAHPs and porcine myocardium. Reproduced with permission from [147]. Copyright 2023, Springer Nature Limited. (D) Direct adhesive attachment achieved by a BASC channel and a wet tissue surface. The double-network design of the BASC contains a semiconducting polymer and an adhesive polymer that achieves covalent bonding with tissue surfaces. (E) Shear strength of the adhesion between a fully bioadhesive OECT on the pig muscle tissue. (F) Conformable and stable contact of bioadhesive OECT enables continuous ECG signal recording than nonbioadhesive OECT [78]. Copyright 2023, AAAS. (G) Schematic illustration of the PHB layer for establishing a robust biointerface between the wireless sensors and biological tissues. Reproduced with permission from [148]. Copyright 2024, Wiley-VCH.

In terms of the dynamic process of wet adhesion, Yu et al. [147] fabricated a chronological adhesive cardiac patch (CAHP) crosslinked by functionalized polyaniline (f-PANi) and polyvinyl alcohol (PVA). The dynamic covalent borate ester bonds and noncovalent hydrogen bonds among them ensure the in situ gelation mechanism and further benefit internal cohesion and interfacial interlocking to firmly anchor with the myocardium (Fig. 4A). CAHP demonstrates an enhanced mechanical interlocking adhesion with tissue by penetrating the wet epicardium and forming tiny and dense pore channels at the tissue interface (Fig. 4B and C). Bioadhesive motifs can also be incorporated into polymer brush for wet tissue adhesion. By means of a polyethylene backbone with long linear side chains, carboxylic acid (COOH) and NHS ester show a synergistic effect in bioadhesive polymer (BAP) [78]. Specifically, COOH groups absorb water for temporarily cleaning and forming electrostatic interactions with tissue surfaces, while NHS covalently bonds with the primary amine groups on the tissue surface. An intrinsic bioadhesive polymer semiconductor (BASC) film was developed by crosslinking with semiconducting polymer poly(3,3′-bis(2-(2-(2-methoxyethoxy) ethoxy) ethoxy)-2,2′:5′,2″-terthiophene) [p(g2T-T)], whose interfacial toughness was significantly higher than that of merely p(g2T-T). The soft and bioadhesive BASC film provides a direct attachment to tissues without gaps and high impedance. Organic electrochemical transistors (OECTs) equipped with BASC were fabricated for electrophysiological recording (Fig. 4D). They have a stable and conformable contact with isolated rat-heart surface even bearing mechanical perturbation, while nonbioadhesive OECT was detached from heart and ECG recording was ceased (Fig. 4E and F). Very recently, a polymer hydrogel bioadhesive (PHB) consisting of poly(2-hydroxyethyl methacrylate-*co*-*N*-vinylpyrrolidone) [poly(HEMA-NVP)] hydrogel layer for swelling-resistant and PAA-NHS brush for adhesive was reported [148]. The laminated PHB formed conformal interfaces with PLGA and porcine tissue, contributed by noncovalent interactions (i.e., hydrogen bonding and electrostatic interactions) and covalent bonding between the NHS (hydrogel adhesive side) and amine moieties (tissue side) (Fig. 4G). Furthermore, the PHB was integrated with a wireless pressure device for intracranial pressure (ICP) monitoring. The robust interface supports a more widely sensing range (0 to 40 mmHg) and higher sensitivity (1 MHz/mmHg) of ICP than sutures, which can be potentially used for real-time physiological and clinical investigations. To sum up, motifs with both physical or chemical interaction with tissue can both enhance the adhesive performance. They can drive bioelectronics anchored tightly to neighboring tissues.

Topological modification is gaining more and more concerns for wet tissue adhesion, owing to its high efficiency in building and enhancing interface interaction via topological interlock or entanglement [23,149,150]. Tissue topology such as pore size, wrinkles, and roughness guided the adhesive architectural design. In addition, inspired by biological wet adhesive surfaces such as octopus, gecko, and Boston ivy, a series of biomimetic structures have emerged [151]. Suckers, polygonal patterns, nanopillar arrays, hook arrays, seta arrays, and so on have been reported with enhanced adhesion force than the flat surface. In terms of topological entanglement, cellulose, alginate, and chitosan were employed as stitch polymers to penetrate into the 2 adherends [152]. Once triggered by pH, a stitch network formed and strong adhesion was achieved among wet materials. With the same strategy, polyacrylic acid (PAA), PAM, poly(n-isopropylacrylamide) (PNIPAM), and cyanoacrylate polymers were also proved to have the potential for topohesive [108,153]. The synergistic effect of combined surface chemistry and topography modification has been confirmed, e.g., a stitch-bonding strategy proposed by Lu and colleagues [23]. Dopamine-grafted PAA and NaIO_4_ were used as glue polymer and the trigger, respectively, for intermolecular crosslinking, demonstrating universal adhesion performance (adhesion energies, ~200 to 400 J/m^2^) to a series of representative adherends [glass, aluminum oxide (Al_2_O_3_), PDMS, porcine liver, and skin]. Till to now, most works about topography modification for better tissue adhesion were presented in materials level instead of device. Thus, it is urgently demanded to integrate novel materials and topological design with bioelectronics to facilitate its practical application in dynamic environment. Hydrogel is a promising platform to eliminate the gap between materials and devices. Recently, Liu et al. [154] reported a stickleback fish-inspired Janus zwitterionic hydrogel patch with microstructures to minimize the likelihood of slippage and detachment from the cecum surface. The concept Janus hydrogel is very suitable to provide specific bioadhesive layers for bioelectronics. We inferred that the combination of Janus hydrogel with bioelectronics may promote the application of topological modification in bioelectronics.

#### Bioactive materials for integrated bioelectronics with multifunctionalization

**Fig. 5.**
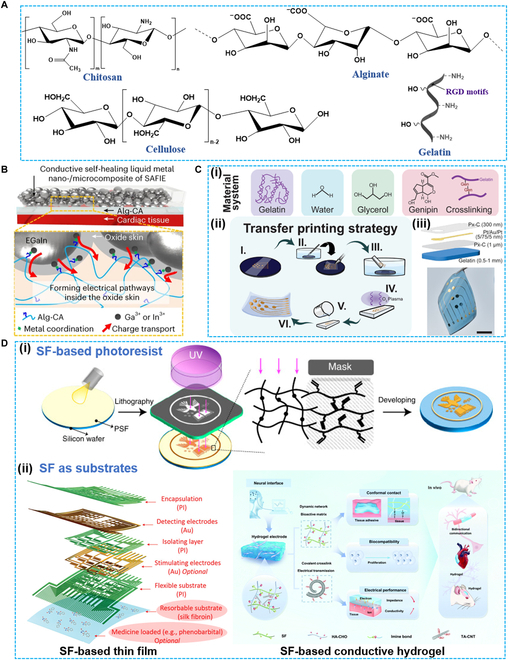
Bioactive materials. (A) Chemical structures of representative bioactive materials for bioelectronics. (B) Sutureless bioelectronic (SAFIE) with Alg-CA for conformal adhesion with heart. Reproduced with permission from [166]. Copyright 2023, Springer Nature Limited. (C) Genipin cross-linked gelatin thin film (i) as electrode substrate via (ii) aqueous-phase transfer printing strategy and (iii) exploded view of the thin-film electrode arrays. Reproduced with permission from [171]. Copyright 2023, Wiley-VCH. (D) SF-based bioelectronics where SF was employed as (i) photoresist (reproduced with permission from [246]; Copyright 2020, National Academy of Science) and (ii) substrates including SF-based thin film (left) (reproduced with permission from [153]; Copyright 2019, Wiley-VCH) and SF-based conductive hydrogel (right) (reproduced with permission from [173]; Copyright 2022, Royal Society of Chemistry).

Although the mechanism is unclear, bioactive materials are promising since they provide multifunction, including anti-immunity, antioxidant, and anti-inflammation, to implant bioelectronics that favor the long-term implantation. Polysaccharides and proteins are the main components of ECM, which have been extensively investigated and applied in biomedicine with or without chemical modification [155]. To improve the soft tissue integration, polysaccharides or protein-based bioelectronics have emerged. Fig. 5A shows the chemical structure of representative bioactive materials for bioelectronics. Chitosan is a polyanion polysaccharide that comes from shellfish waste containing -NH_2_, -OH, and -NHCOCH_3_ groups. Chitosan and its derivates have been reported with antibacterial, antioxidant, immune, and antitumor properties [156]. The chitosan-based film has been employed to fabricate bioprotonic field-effect transistor (proton mobility of ~4.9 × 10^−3^ cm^2^/(V·s)), internal ion-gated organic electrochemical transistor (IGT) and nonvolatile memory device as channel materials, as well as optoelectrode arrays and neural probes as encapsulation materials [157–159]. With strong intramolecular hydrogen bonding, chitosan is a promising candidate to fabricate anti-swelling hydrogel. Qi and colleagues [160] reported a nonswelling, wet adhesion hydrogel (PAACP) composed of poly(vinyl butyral), acrylic acid, gelatin, and chitosan-grafted *N*-acetyl-l-cysteine for bioelectronics. After implantation for 14 d in vivo, PAACP preserved the tough adhesion performance, supporting the long-term monitoring of electrode array and strain sensor. Cellulose is the most abundant biopolymer on earth, which can be extracted from forests or secreta by microbes [161]. Similar to chitosan, cellulose with many -OH groups possesses strong intramolecular hydrogen bonding, making it promising to fabricate tough film and hydrogel. Compared with other biomaterials, cellulose is less immunogenic but is biodegradable since it is plant-originated. Cellulose nanocrystals (CNCs), cellulose nanofibers (CNFs), and BC, which have high aspect ratio, are the main derivates of cellulose, and they have been used as substrates for several bioelectronics (e.g., neural probe, conductive biopatches, and biosensor) [162]. Alginate is a soluble, brown seaweed-extracted polysaccharide with alternated mannuronic acid (M unit) and guluronic acid (G unit). It can be crosslinked by divalent cations (e.g., Ca^2+^, Cu^2+^, and Sr^2+^) and forms a unique “egg-shell” structure. Alginate has been reported to possess multiple bioactivities (e.g., antioxidation and anticoagulation), which provide many benefits for its biomedical application, such as cargoes for drug and cell delivery and scaffolds for tissue engineering [163–165]. Complexed with conductive filler [MXene, graphene oxide (GO) CNTs, silver nanowires (AgNWs), PEDOT:PSS, PANi, and so on], alginate-based conductive hydrogel has drawn a lot of attention owing to its soft and stretchable advantages [58]. Fig. 5B displays a sutureless bioelectronic (SAFIE) composed of 3 layers: an ionically conductive tissue adhesive, a viscoelastic networked film, and a fatigue-resistant conducting composite (E-SHN) [166]. Alginate modified with dopamine (Alg-CA) was coated and penetrated into E-SHN to provide an instantaneous (<60 s), robust (adhesive strength ~ 7.2 kPa), stretchable (strain ~ 660%), and conformal adhesion with heart. Moreover, metal coordination between carboxylate/catechol groups in Alg-CA and Ga^3+^ and/or In^3+^ of EGaIn results in an electrical pathway, which benefits the durable and real-time cardiac monitoring. Polysaccharides with different mechanical and biological features can work together as bioelectronic–tissue interface materials (BTIM), e.g., the formula consisted of bioresorption polyethylene glycol-lactide acid diacrylate (PEG-LA-DA), alginate, and chitosan. PEG-LA-DA possesses photopolymerization nature that accounts for sol–gel transformation, alginate forms ionic network with Ca^2+^ to enhance the electrical conductivity, and chitosan mixed with coupling reagents functions as a primer for the tissue surfaces and forms a covalent bond (-CO-NH-) as well as physical chain entanglement with alginate. The synergistic effect of them enables a high-adhesion and low-impedance BTIM for optogenetic electronic platforms and electrical cardiac pacing.

Collagen, gelatin, and silk are the main natural proteins that have been reported for bioelectronics application, specifically as encapsulation layer or substrate of implantable bioelectronics [167]. Collagen and gelatin extracted from connective tissues of pig and bovine species, fish species, and so on have distinct amino acid composition and physical and chemical properties. The pig and bovine species are mostly adopted to construct film and hydrogels due to their easy chemical modification, better processability, and wide availability. Collagen with 3 long helix-shaped chains is water-insoluble, while its next hydrolysis product gelatin presents a temperature-controlled solubility [168]. In addition to biocompatibility and biodegradability, gelatin has cell binding motifs [arginine–glycine–aspartic acid (RGD) sequence] in nature, making it appealing in the biomedical field. Gelatin-based bioelectronics has demonstrated several advantages such as flexibility, self-healing, biodegradability, self-adherence, and recyclability [23,169]. Recently, Zhang et al. [170] reported a 10-μm-thick gelatin-based hydrogel skin sensor reinforced by PU nanomesh. The ultrathin sensor has high stretchability (strain ~ 700%), high adhesion (adhesion energy ~ 176.8 μJ/cm^2^), low impedance (~31.3 kΩ), and good biocompatibility, which can be used for long-term monitoring of electrophysiological signals without causing skin irritation, itches, or inflammation. As a common food additive, gelatin can also be fabricated into ingestible soft bioelectronics. After chemical crosslinking by genipin and changing the solvent from water to water:glycerol, the resulting soft gelatin hydrogel (elastic modulus ~ 33.32 kPa; toughness ~ 39.32 J/m^3^) was employed as the substrate (Fig. 5Ci) [171]. To integrate with multielectrode arrays, a wet transfer printing process was conducted (Fig. 5Cii). Parylene-C and thin-film metal-based multielectrode arrays were detached from rigid silicon wafers via capillary forces of water and transferred to another clean substrate. After being cleaned by plasma, the gelatin precursor was drop-casted on the back surface of the electrode arrays. The lamination structure and optical image of the transferred device are shown in Fig. 5Ciii. Furthermore, the device was applied as impedance sensor for epithelial barriers via electrochemical impedance measurements.

Silk fibroin (SF) that originated from silkworm silk has been applied in our daily life since ancient times. In recent decades, SF-based biomaterials (e.g., hydrogel, film, scaffolds, and NPs) are drawing great attention for their good in vivo biocompatibility, biodegradability, cell affinity (RGD sequences), tunable biomechanical properties, and a variety of chemical modifications [e.g., SF-methacrylate (SFMA)] [172]. With high compliance with the nanofabrication process, SF-based flexible bioelectronics is blooming. Fig. 5D presents the most typical application of SF in bioelectronics: SF-based photoresist, where SF functions as the substrate in the form of thin film and bulk conductive hydrogel. By means of chemical modification, SF can be developed to be photoactive, which has been adopted for high-resolution micropatterns via photolithography. By mixing with electroactive component, SF-based bioelectronics was constructed. Transfer printing is another method to construct SF-based thin-film devices. As shown in Fig. 5Dii, Mao and colleagues [153] reported an SF-supported/delivered soft bioelectronics for the investigation of spatiotemporal epileptiform activities and multimodal neural encoding/decoding. The biocompatible SF substrate can not only spontaneously form conformal coupling with the cortical surface but also work as vehicle for in vivo drug delivery. SF-based conductive hydrogel can be constructed by introducing electroactive materials (e.g., PEDOT, CNT, and MXene). On the left of Fig. 5Dii, it demonstrates a mechanically adaptive hydrogel crosslinked by SF and aldehyde-hyaluronic acid (HA-CHO) for high-efficiency neural activity recording [173]. The dynamic crosslink network provides a soft and tissue-compatible hydrogel for immune modulation, and tannic acid–CNTs were introduced to bring conductivity. The soft electrode can record epicardial ECG signals with a higher SNR than rigid Pt electrode (37 and 15, respectively) and causes fewer immune responses and biological toxicity after implantation.

Apart from biopolymer, nanomaterials such as PDA NPs, graphene, and MXene have been reported to possess antioxidizing activity; thus, incorporation of them would benefit the in vivo performance of implantable bioelectronics. Dopamine has demonstrated its outstanding performance in alleviating the inflammatory response in addition to bioadhesion. In a brain injury model, bioelectronics containing dopamine suppresses inflammation and prevents further fibrosis by regulating the activity of macrophages. To sum up, bioactive materials are mainly employed as substrates for bioelectronics to enhance their biocompatibility. Although their bioactivity has been verified in other fields such as tissue engineering and drug delivery, the role in bioelectronics needs more profound investigation.

## Application of Implantable Bioelectronics

### Monitoring and sensing of physiological signals

With the advancement of implantable sensors, various physiological signals have been acquired for healthcare monitoring, including electrophysiological signals [electroencephalography, electrocardiography, electromyography (EMG), etc.], temperature, pH, and pressure [10,174–177]. This section will focus on devices for monitoring electrophysiological signals, following the order of electroencephalography, electrocardiography, and EMG.

#### EEG monitoring

Brain signals reflect neuronal activity in the brain, and obtaining high-fidelity, high-resolution brain signals is crucial for brain research and for diagnosing neurological diseases [67,178,179]. Depending on their location, electrodes record EEG on the scalp, ECoG on the surface of the cortex using subdural grid electrodes, and localized field potentials (LFPs) in the brain with small-sized electrodes [180]. Advances in signal recording devices aim to capture electrophysiological signals with high quality. Conventional implantable brain signal acquisition uses rigid implantable electrodes, of which silicon-based Utah electrodes and Michigan electrodes fabricated by microelectromechanical system (MEMS) fabrication are typical representatives, which can record multipoint signals and local field potentials [181]. Utah electrodes have the advantages of high channel count, safety, and excellent short-term recording stability [182]. Whereas Michigan electrodes have high design flexibility and high spatial resolution [183]. However, there is a mechanical mismatch between the rigid probe and the soft brain tissue, which causes an immune response in the surrounding neural tissue upon implantation and leads to tissue scarring in long-term experiments, thus affecting the long-term stability of the device [12,184]. Therefore, flexible probes based on polymer substrates have been studied to reduce tissue responses.

**Fig. 6.**
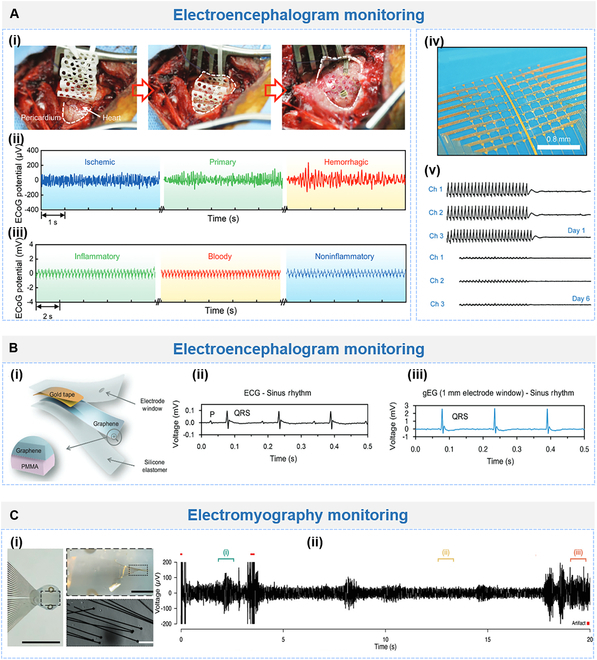
Flexible electrodes for electrophysiological signal recording and monitoring. (A) (i) Rigidity switch and shape recovery of the electrode during minimally invasive surgery. (ii) Recorded ECoG signals and (iii) ECG signals. Reproduced with permission from [174]. Copyright 2023, Wiley-VCH. (iv) Optical image of the OECT array. (v) μ-ECoG signals recorded by 3 channels of the transient OECT array on days 1 and 6. Reproduced with permission from [33]. Copyright 2023, Wiley-VCH. (B) (i) Structure of the electrode featured by submicrometer-thick graphene. (ii) ECG signals recorded by commercial Ag/AgCl electrodes and (iii) graphene electrodes. Reproduced with permission from [53]. Copyright 2023, Wiley-VCH. (C) (i) Tetrode-like arrangement of microelectrodes. (ii) Electrode recordings of EMG signals on day 1. Reproduced with permission from [193]. Copyright 2023, Wiley-VCH.

Recently, pioneering advancement has been made in flexible probes. Jiao et al. [174] introduced a multimodal device employing shape memory polymers. This device, with controllable stiffness and shape, integrates multiple sensors in the rigid state, enabling minimally invasive implantation in the soft state and conformal adherence to tissue after shape recovery (Fig. 6Ai). The impedance modulus of the integrated electrode at 1 kHz was 1.35 kΩ, enabling the recording of ECoG and ECG signals in vivo (Fig. 6Aii and iii). The recorded ECoG signals had similar waveforms and spectra to those of commercial electrodes. The characteristic ECoG signals of epilepsy were recorded near the frequencies of 12.5 and 25 Hz. Furthermore, it diagnosed ischemic, primary, and hemorrhagic epilepsy by combining ECoG and temperature signals and identified inflammatory, hemorrhagic, and non-inflammatory pericardial effusions using collected ECG signals along with pH signals.

In order to obtain signals with higher resolution, minimizing the size of the electrode has been a trend in device design. Viana et al. [185] presented a graphene-based nanoporous thin-film microelectrode (EGNITE, 25 μm in diameter) and integrated a 64-channel microelectrocorticography (μ*-*ECoG) array of EGNITE, with a pitch of 300 μm and a thickness of about 12 μm. These electrodes exhibit low impedance (25 kΩ) and high charge injection (3 to 5 mC/cm^2^). Positioned on the auditory cortex, the electrodes record neural signals with high fidelity and spatial resolution, achieving an SNR greater than 10 dB for the local field potential signal.

Moreover, sensitivity is also required for precise neural recording. Liu et al. [186] developed a neuroelectrode based on nano-enzymes, which utilized quantum transport and biocatalytic processes to achieve both electron and ion conductive at the electrode tissue interface. The SNR of single-neuron recording in vivo can reach 14.7 dB, and the impedance of the nano-enzyme electrode is ^1^/_26_ of conventional silver electrodes. Additionally, the SNR for epilepsy detection is 3.2 times higher than that of silver electrodes, and it is at least 9.3 times more sensitive than the silver electrode at the second month after implantation, and the nano-enzyme electrode exhibits good sensitivity stability. Wu et al. [33] presented a multichannel neural interface for high-fidelity mapping of μ-ECoG signals (Fig. 6Aiv). The OECT-based network with 200 × 20 μm^2^ conductive channel size enabled stable recording at 100 sites, which realized the SNR up to 37 dB for in vivo μ-ECoG signals. Furthermore, the biodegradable device can be degraded into micro-nanoparticles after immersion in PBS, which avoids the risks associated with secondary surgery. One week after implantation, the recorded μ-ECoG signal was attenuated due to the degradation of PEDOT:PSS (Fig. 6Av).

#### ECG monitoring

On the other hand, the ECG reflects the contraction and diastole of heart muscle, aiding in arrhythmia prevention and diagnosing cardiovascular diseases (CVDs) [1,48]. Myocardial tissue has a Young’s modulus of around 10 kPa, exhibits regular contraction and diastole, and requires efficient blood supply for oxygen and nutrients [187]. Therefore, in order to achieve a seamless and stable interface, the bioelectronic device should be soft enough and have high stretchability, toughness, and conductivity simultaneously, as well as wet adhesion properties [188]. At the same time, the materials used should be biocompatible and able to reduce immune response and promote the growth of cardiomyocytes in order to record ECG signals in real time, track cardiac function, and promote the growth and healing of damaged myocardial tissue [156,189]. In consideration of ultrasoft materials, Sim et al. [18] reported an submillimeter thickness epicardial patch, which contained PDMS substrate and encapsulation, conductive rubbery paste, and PDMS conductor with embedded AgNWs, possessing inherent porcine heart tissue-like modulus and conductivity. Besides, many efforts have been devoted into balancing the mechanical and electrical properties of the bioelectronics. For instance, Wang et al. [19] introduced an intrinsically stretchable patch for conformal adhesion on the beating heart. The 16-channel device composed of the SEBS substrate, liquid metal interconnects, and CNT nanocomposite electrodes reached ultrahigh elongation up to 400% and exhibited excellent conductivity of 3.8 × 104 S/cm. Meanwhile, reliable wet adhesion of the device is necessary for the sensitivity and stability of the signal. Yu et al. [147] proposed a CAHP whose precursor solution contained amphiphilic side chain-modified polyaniline (f-PANi), which rapidly absorbed anti-adhesive pericardial fluid, actively diffused, and autonomously formed cross-links and mechanical interlocks at the interface, allowing the patch to conformally attach directionally to moist and curved epicardial membranes with an interfacial toughness of up to 443.4 J/m^2^. Lin et al. [53] employed a submicrometer-thick graphene bio-interface on the heart surface for ECG signal acquisition and stimulation (Fig. 6Bi). The electrocardiogram (gEG) R-waves recorded with graphene electrodes exhibit good temporal correlation with conventional far-field ECG R-waves acquired with commercial Ag/AgCl electrodes and effectively attenuate the electrical noise from the mechanical beats of the heart (Fig. 6Bii).

#### EMG monitoring

Myoelectric electrodes are capable of extracting electrical signals from muscles and of great importance in prosthetic control [190]. First, obtaining high amplitude and sustainable EMG signals can better reflect the fatigue level and excitation state of the muscles and enhance the function of the prosthesis, which has become one of the goals for electrode development. The PEDOT-coated stainless steel electrodes for EMG recording were developed. Using galvanostatic electrodeposition, PEDOT doped with lithium perchlorate was coated onto stainless steel with a diameter of 76.2 mm. Electrodes were inserted into the mice neck muscles to measure EMG activity for 6 weeks. Higher SNR and amplitude were attained with PEDOT-coated electrodes in comparison to bare electrodes, thus improving signal quality. Second, in order to obtain localized muscle potential signals, miniaturized electrodes have been developed to obtain higher spatial resolution EMG signal recordings [191,192]. However, miniaturization reduces the contact area of the electrode with the muscle tissue, resulting in a rise in contact impedance. To address this issue, Boys et al. [193] mimicked the structure of muscle fibers and designed myoelectric electrodes containing 32 electrode sites. The arrangement of the electrodes was tetrode-like, and the diameter of the recording pads was 20 μm (Fig. 6Ci). In terms of material design, the recording electrodes were gold electrodes with PEDOT:PSS coating with parylene-C encapsulation. The lead arrays were formed in a fibrillar collagen gel by injection of type I collagen extracted from rat tail tendon. The average impedance of the electrodes was measured to be 48.9 kΩ at 1,000 Hz, allowing the recording of intramuscular EMG with high spatial resolution (Fig. 6Cii).

### Modulation of physiological and pathological conditions

Bioelectronics serving as implantable medical device can modulate the conditions around implant sites or host by delivering drugs, namely, the bioelectronic drug delivery systems (bEDDSs) [41,194]. Here, we focus on the implantable bEDDS, which works in vivo instead of in vitro (transdermal). For instance, heparin-doped polypyrrole electrodes (PPy/Hep) can deliver anti-inflammatory cytokines (IL-4) in vivo that mitigate FBR around implants [30]. Local delivery of inhibitory neurotransmitter γ-aminobutyric acid (GABA) in a neuropathic pain (NP) model via organic electronic ion pump (OEIP) can significantly decrease the pain response after implantation for 2 d [195]. Microneedle-integrated bioelectronics with localized delivery of theragnostic NPs and drugs has been used for precise treatment of lesions [196]. However, active control of drug release along with implant bioelectronics needs further integration of material science and bioelectronics.

**Fig. 7.**
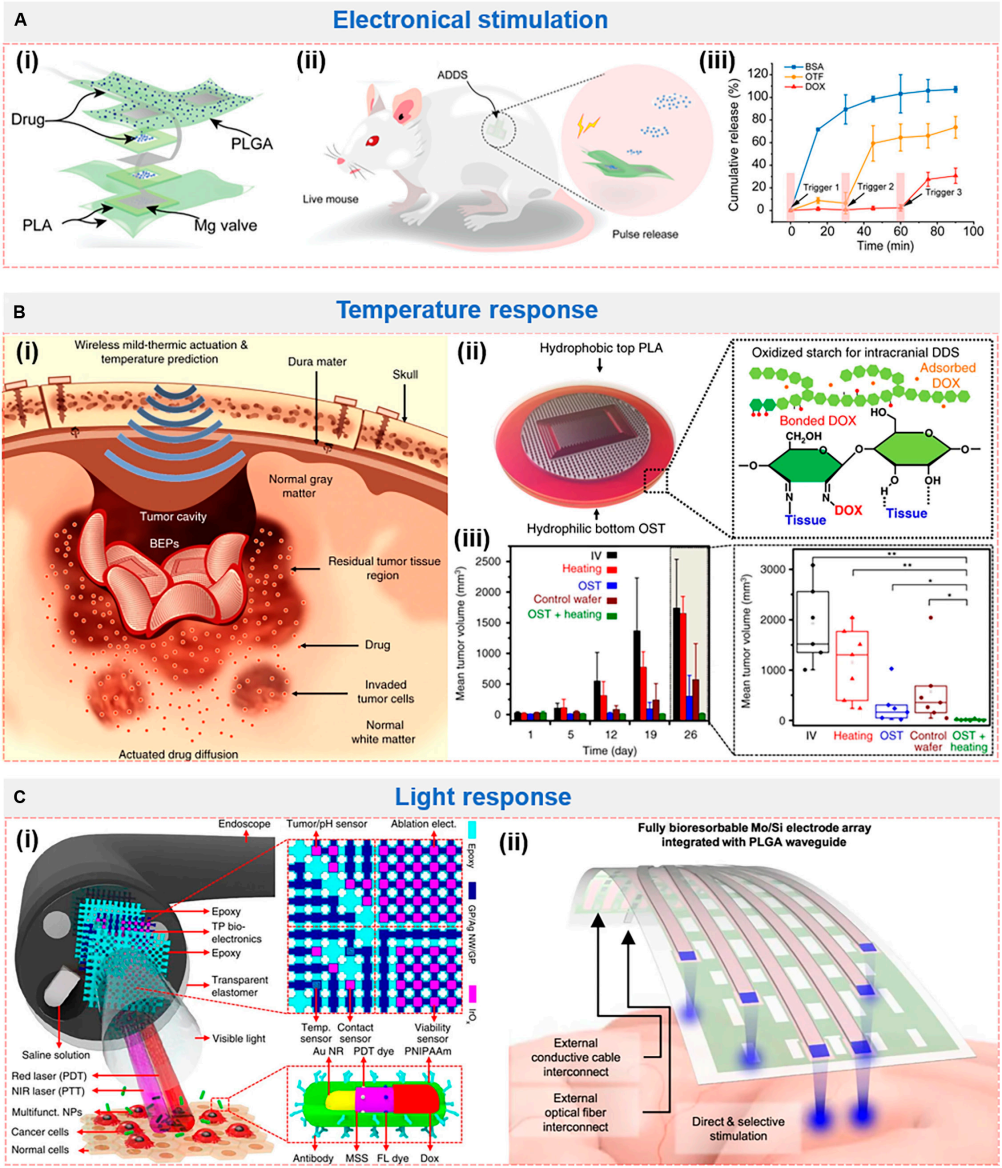
Implantable bioelectronics with DDS application triggered via (A) electronical stimulation, (B) temperature, and (C) light. (A) Biodegradable multifunctional active-controlled drug delivery system (ADDS) (i) for pulsatile (ii) and sequential (iii) release of multiple drugs. Reproduced with permission from [34]. Copyright 2023, Wiley-VCH. (B) Bioresorbable electronic patch (BEP) with wireless mild-thermic actuation (i) consisting of OST to load DOX (ii) for efficient treatment of brain tumor (iii). Reproduced with permission from [198]. Copyright 2019, Springer Nature Limited. (C) Multifunctional endoscope system combined with PDT, PTT, and chemotherapies for colon cancer treatment (i). Reproduced with permission from [201]. Copyright 2015, Springer Nature Limited. (ii) Bioresorbable neural implant system for simultaneous electrophysiology and optogenetics stimulation. Reproduced with permission from [205]. Copyright 2024, Springer Nature Limited.

Similar to DDS, biodegradable or smart responsive materials (thermal, light, electrical, and so on) are promising candidates for drug storage and release system. PLGA, a Food and Drug Administration (FDA)-approved biodegradable polymer, was frequently employed to load drugs. For example, an implantable and bioresorbable microneedle (IBMN) device was fabricated by combining wireless electrostimulation (ES) and drug delivery, which had microneedles made of PLGA to load and release drugs [197]. The loaded aspirin and ibuprofen can continuously be released over 15 d, along with hydrolysis and erosion of PLGA. Magnesium (Mg) can react with surrounding biofluids (Mg → Mg^2+^ + 2e^−^) and degraded gradually [14]. Such an electrochemical etching process was employed by using Mg as gate to control the release of liquid drug storage in a bioresorbable polyanhydride polybutanedithiol 1,3,5-triallyl-1,3,5-triazine-2,4,6(1*H*,3*H*,5*H*)-trione penteonic anhydride (PBTPA) [33]. Under the similar mechanism, a Mg-gated biodegradable multifunctional bioelectronic system (ADDS) was fabricated (Fig. 7A) [34]. The biodegradable ADDS consisted of a drug-carrying PLGA membrane, tandem Mg valves, and poly(lactic acid) (PLA) membrane aligning in the *z*-axis direction for drug storage and pulsatile/sequential deliver (Fig. 7Ai). The flat soft ADDS can be bent/folded into tubular, which meets the application of subcutaneous or intravascular drug delivery (Fig. 7Aii). Under electronical stimulation, Mg valve was corroded and drugs encapsulated in the PLA membrane started to release in a controlled and sequential manner (Fig. 7Aiii). Drug delivery to local brain tissues that bypasses the blood–brain barrier (BBB) is important for malignant brain disease [glioblastoma (GBM)] treatment [40]. A chemo- and thermo-coupled therapy was carried out via wireless biodegradable electronic patch (BEP) (Fig. 7Bi) [198]. The BEP consisted of 3 components: wireless temperature sensor for thermal control, wireless heater for heat production, and drug reservoir for thermal response. The materials used to construct them were biodegradable PLA, PLGA, Mg, and oxidized starch (OST), ensuring the little potential neurological side effects. Hydrophilic OST displays more sustained drug release than unmodified starch, as well as a conformal contact with brain surgery site (Fig. 7Bii). In a canine GBM model, the group treated with combinate “OST + heating” presents the best therapeutic efficacy (Fig. 7Biii). Thus, apart from the long-lasting doxorubicin (DOX) delivery to the target region, mild-thermic actuation increases drug penetration and thus suppresses tumor growth.

Light-related modulation is gaining more and more concerns in theragnostic recently, e.g., photodynamic therapy (PDT), photothermal therapy (PTT), and optogenetics [39,199,200]. Many conductive nanomaterials (e.g., GO, CNTs, and MXene) can also work as photo-thermal conversion agents, which expands their biomedical applications. Limited by the penetration depth of light, PTT mainly focuses on surface wound management. Li et al. [63] reported a gelatin-oxidized dextran (Gel-ODex) hydrogel-based bioelectronics containing magnetoelectric nanosheets (Fe_3_O_4_-PDA-rGO), which can proceed and monitor the wound healing via photothermal antibacterial and anisotropic sensing. The in vivo delivery of light/photo can realize the application of PDT/PTT in deep tissue of inside organ. An endoscope combined with laser light [near-infrared (NIR), red, and visible light], transparent bioelectronics, associated sensors (temperature, pH, contact, viability sensor), multifunctional NPs [chlorin e6 (Ce6), gold nanorods (Au NRs)], and chemo-drug DOX was fabricated (Fig. 7Ci) [201]. Thermosensitive PNIPAM (upper critical solution temperature ~ 32 °C) was employed to respond to NIR laser and release DOX. The “all-in-one” platform can sense, ablate, and kill tumor cells, and the combination of PDT (ROS), PTT (heating), and chemotherapy performs best in the colon cancer treatment.

Optogenetics is a neuromodulation technique that can manipulate neurons at millisecond-order temporal accuracy through optical stimulation by expressing photoactive microbial opsin proteins in the desired nerve cells [202,203]. A gene-embedded optoelectrode array aiming at neural implantation to enable spatiotemporal electroporation (EP) was fabricated with multifunctions, including gene delivery/transfection, photo modulation, electrical monitoring, and light stimulation of neural tissue [204]. The nanostructural hydrogel consisting of rGO and 3,4-ethylenedioxythiophene-modified amphiphilic chitosan was used as neural interface (called PG) for controllable gene delivery with electrically inductive electro-permeabilization. The in vivo implantation of the “all-in-one” optoelectrode enables targeting and on-demand spatiotemporal gene transfection. Integrated with optical waveguide, photoevoked extracellular spikes were detected on day 7, convincing the success of optical stimulation. Recently, a fully biodegradable opto-electronic neural implant system was developed based on molybdenum/silicon (Mo/Si) electrode array and PLGA waveguide (Fig. 7Cii) [205]. The soft and flexible device caused negligible immune responses and completely dissolved by day 50. Chronic in vivo optogenetics experiment was carried in Thy-1: ChR2 transgenic mice. Induced by optical stimulation with 460-nm pulses, evoked local field potentials were enhanced and exhibited periodicity until day 28, confirming the ability of optogenetic stimulation.

bEDDS device integration relies heavily on the responsive materials for drug storage and release. However, the existing works mainly deliver drugs in a passive way, and the sophisticated materials design and the combination of smart bioelectronics need further exploration. Besides, electrical simulation itself can also play roles in pathological modulation [72,112]. Magnetic resonance imaging (MRI) compatibility transparent PEDOT:PSS-based ECoG grid for NP relief via motor cortex stimulation (MCS) was developed [61]. We would discuss it in detail in terms of deep brain stimulation (DBS) in the next section.

### Tissue engineering

Bioelectronics are appealing in electroactive tissue engineering as electrical stimulation contributes to the regeneration of cells and secretion of cytokines and growth factors [206,207]. Tissues or organs including muscle, heart, brain, and so on that take electronic signals for information transfer have benefited from electronic stimulation for a long time, such as DBS, spinal cord stimulation (SCS), cardiac pacemaker, and electrical muscle stimulation (EMS) [20,206,208]. Besides facilitating repair, implant bioelectronics are also promising in replacing the natural tissue with function reconstruction, e.g., electronic skin (e-skin) [209]. In this section, we mainly focus on the application of implantable bioelectronics in nerve/brain tissue engineering and myocardial tissue engineering.

#### Nerve/brain tissue engineering

**Fig. 8.**
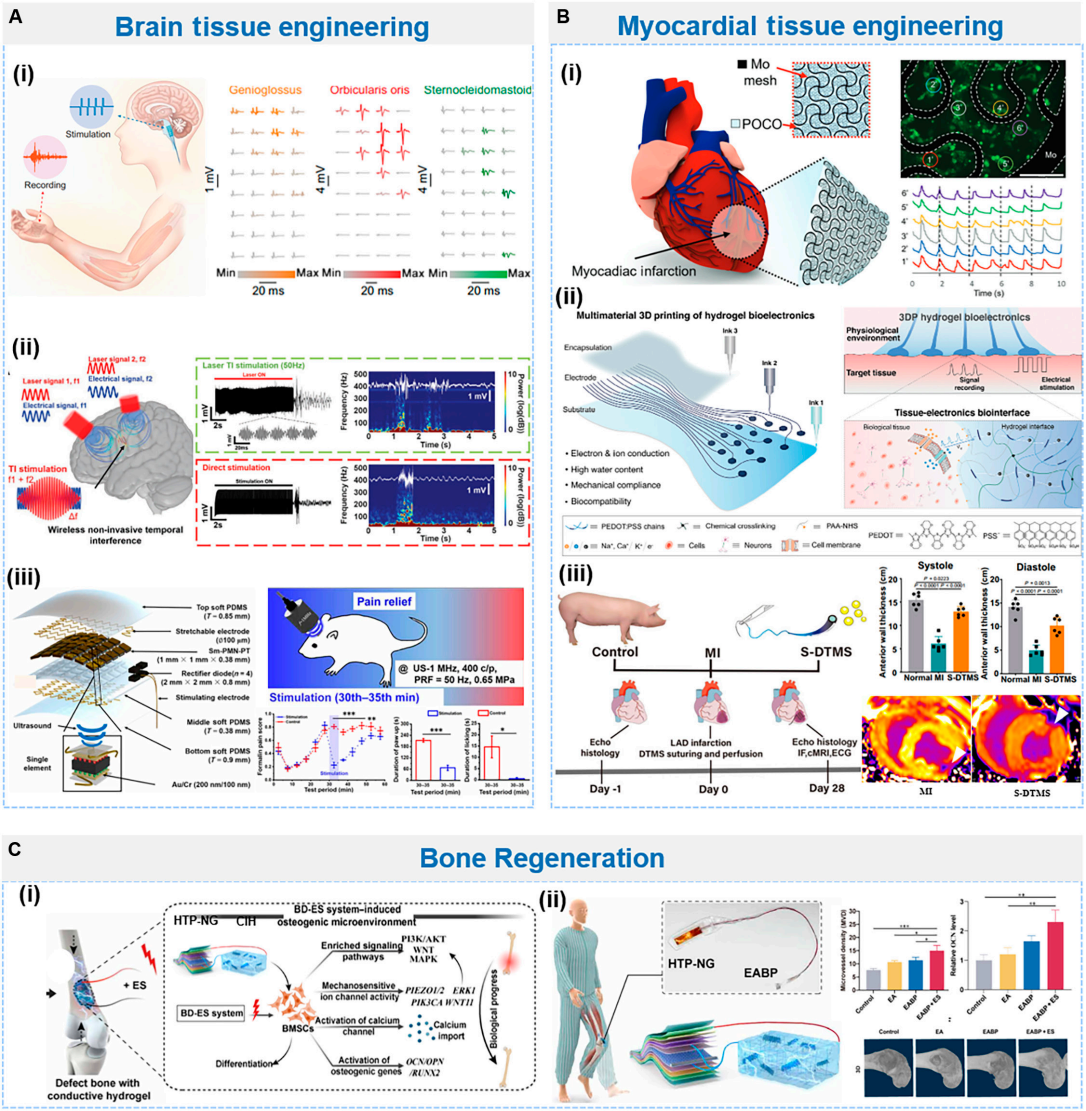
Application of implantable bioelectronics in (A) brain, (B) cardiac, and (C) bone tissue repair. (A) Brain tissue engineering via (i) stretchable multielectrode array for bidirectional electronic stimulation with high precision (left) to evoke and record muscle activities at the tongue, whisker, and neck (right). Reproduced with permission from [55]. Copyright 2022, AAAS. (ii) Laser-driven wireless DBS by TI technology (left) with comparable ability to evoke electrophysiological epileptiform activities as direct DBS (right). Reproduced with permission from [220]. Copyright 2022, Wiley-VCH. (iii) Ultrasound energy-harvesting (Sm-PUEH) device (left) for pain relief via DBS. Reproduced with permission from [200]. Copyright 2022, AAAS. (B) Cardiac tissue engineering via (i) bioresorbable cardiac patches (left) with the ability to facilitate cell proliferation via electronic stimulation (right). Reproduced with permission from [234]. Copyright 2023, Wiley-VCH. (ii) 3D-printed hydrogel electronics for electrophysiological monitoring and electrical modulation. Reproduced with permission from [9]. Copyright 2023, Wiley-VCH. (iii) Microchanneled hydrogel suture to diagnose, treat, and monitor the infarcted heart. Reproduced with permission from [237]. Copyright 2024, Springer Nature Limited. (C) Bone regeneration via (i) Fully implantable battery-free BD-ES system and possible mechanism involved in the osteogenesis process. (ii) Performance of BD-ES system in promoting bone repair via electrical stimulation. Reproduced with permission from [239]. Copyright 2024, AAAS.

Neurodegenerative disease [e.g., Alzheimer disease (AD) and PD] and traumatic brain injury (TBI) result in low quality of life (losing control of recognition and behavior) or longevity [172,193]. The treatment of brain-related diseases is hindered by the complex structure of the nerve network. Fortunately, BCI provides a technology for neurophysiological and neurological disease researches, based on monitoring, recording, decoding, and modulating neuron activities [210]. For example, based on the BCI device, paralyzed patients can restore movement, touch, and speech [210–213]. However, those devices are commonly based on rigid platinum–iridium electrode/probe with risk (e.g., secondary brain injury) for implantable application. Soft bioelectronics with mechanical match with brain tissues are desirable in neuromodulation. Fig. 8Ai illustrates stretchable organic bioelectronics based on the topological supramolecular conducting polymer network (PR) for both recording and modulation [55]. It allows direct photopatterning at cellular-level feature sizes and withstands over 100% strain without crack. The high conductivity of PR-based microelectrode array ensures stable electrophysiological monitoring and precise neuromodulation at low-voltage (100 μV) electrical stimulation. Upon localized brainstem stimulation, evoked muscle activities at tongue, whisker, and neck were recorded with distinct signals.

Direct electronic stimulation is also facing several limits, such as complex circuitry, large body, and high-power consumption [214,215]. To improve the accuracy and convenience of neuromodulation, laser or ultrasound (US)-based DBS has also been presented [216–218]. For instance, multifunctional neural probes with integration of optogenetics and electrophysiology for optogenetic manipulation are equipped with microscale light-emitting diodes (μLEDs), optic fiber, or adeno-associated virus [82,200,202]. Hong and colleagues [219] reported macromolecular infrared nano-transducers for deep brain stimulation (MINDS). In the irradiation of second near-infrared (NIR-II; wavelength, 1,000 to 1,700 nm), MINDS can generate heat (photothermal conversion efficiency ~ 71%), followed by the activation of a nonselective cation channel with a high permeability to calcium (TRPV1). MINDS were injected into the mouse hippocampus, motor cortex, and ventral tegmental area (VTA), and under NIR-II illumination, and TRPV1 was activated with minimal thermal damage. Fig. 8Aii displays the application of the temporal interference (TI) technique in wireless DBS based on organic electrolytic photocapacitors (OEPCs) that possess optical-electronic transfer ability [220]. The OEPCs were implanted in the cortex, while the neuronal response evoked by the TI technique was comparable to direct deep brain implanted electrodes.

US containing mass energy can be transformed into electric power via the piezoelectric effect [216,221]. Implantable high-performance hydrogel nanogenerators (HENGs) have been reported to be capable of stimulating vagus nerves for anti-inflammatory therapy [222]. Short-circuit current (1.6 mA) with comparable output to commercial bulky neurostimulators was induced by US (power density of 0.3 W/cm^2^). The in vivo application of HENG shows great effectiveness in the inhibition of pro-inflammatory cytokines. Likewise, the piezoelectric ultrasound energy-harvesting (PUEH) device can also be used for DBS (Fig. 8Aiii) [200]. Flexible PUEH was constructed by Sm-doped Pb(Mg_1/3_Nb_2/3_)O_3_-PbTiO_3_ (Sm-PMN-PT) single crystal with high piezoelectric coefficient (4,000 pC N^−1^). Under US, output power can be up to 280 mW so that DBS can be realized. In vivo rat electrophysiological experiments under anesthesia and behavioral experiments further confirmed that the produced current by PUEH activated the periaqueductal gray (PAG) to reach the aim of analgesia. Recently, soft piezoelectric-triboelectric hybrid nanogenerators (PTNGs) were fabricated by employing trimethyl chloromethyl ammonium trichloro manganese (TMCMMnCl_3_) molecular crystals [223]. With programmable US pulses, the soft PTNGs generate a stimulation current of ~0.9 mA. The biocompatible PTNG device was implanted in Parkinson’s rat where US stimulation was performed. Results of electrophysiological study, immunohistochemical staining, and behavior test confirm that the soft high-performance bioelectronic device can meet the requirement of DBS electroceutical application.

#### Myocardial tissue engineering

CVDs are becoming the main diseases that endanger human health [224]. Chronic monitoring of CVD-related parameters such as heart rate and blood pressure can predict the health state of cardiovascular system [225]. Under the malignant conditions such as atrioventricular (AV) block and myocardial infarction (MI), therapeutic interventions are necessary [226]. Cardiac pacemaker consisted of pacing leads (electrodes), and pulse generator is a common device with history over 60 years that aimed for cardiac rhythm dysfunction. However, implantation of stiff pacing electrodes causes several complications including detaches or fracture of electrodes and infection, hematoma, as well as erosion into the tissue. In addition, energy supply of cardiac pacemaker limits its permanent application. Thus, the next generation of pacemakers with battery-free, wireless, and good biocompatibility is urgently needed. Until now, biomaterials that can regulate cardiac microenvironment (e.g., scavenging ROS, suppressing inflammation, and promoting angiogenesis) and contribute to the regeneration of fibrotic tissue with satisfying electrical conduction and mechanical toughness are emerging [227–229]. Among them, soft and stretchable bioelectronics that offer real-time dynamic monitor and simultaneous electrocoupling therapy stands for the smart and efficient platform [230]. For instance, stretchable nanocomposite nanowires containing Ag–Au–Pt core–shell–shell structure and Pt NPs, cardiac peacemakers based on implantable triboelectric nanogenerator (TENG), acoustic energy transfer and communication device (AECD), transparent microelectrode arrays, and so on have been reported [231–233]. Recently, bioresorbable metal Mo was integrated with the poly(1,8-octamethylene-citrate-co-octanol) (POCO) elastic polymer substrate and bioadhesive PEG-LA-DA hydrogel as elastic cardiac patch (BCEP) for MI therapy (Fig. 8Bi) [234]. The sophisticated Mo mesh and elastic POCO can withstand elastic deformation of ~15% (13% of cardiac surface), and PEG-LA-DA hydrogel served as adhesion layer with low impedance of BCEP and tissue (peeling force ~ 0.3 N, resistance ~ 14 kΩ). The biocompatible BCEP facilitates human to induce pluripotent stem cell-derived cardiomyocyte (hiPSC-CM) maturation with enhanced cell contraction behaviors for the conductive path of Mo mesh, indicating their potential application in heart function reconstruction.

Hydrogel-based bioelectronics possesses unique advantages in MI repair for its ECM-like “soft and wet” properties and multicompatibility to bioactivates [60]. Curcumin is a molecule that has been reported to possess anti-inflammatory and CVD treatment potential. However, its bioavailability is limited by poor water solubility. Hou and colleagues [94] fabricated curcumin@polydopamine nanoparticles (CUR@PDA NPs) with typical bilayer core–shell structure and introduced them into conductive hydrogel (called PCP) crosslinked by gelatin and PAM for synchronous MI repair and real-time monitoring. The PCP hydrogel is hyperelastic with negligible hysteresis (elongation at break ~1,300%) and mechanoelectrical sensitivity (37 ms) for cardiac signal monitoring. In addition, PCP hydrogels inherit the bioactivity of CUR@PDA NPs, which benefits MI repair by regulating the inflammatory microenvironment, promoting angiogenesis, and reducing myocardial fibrosis. In MI model based on Sprague–Dawley rats, PCP hydrogel implanted groups showed the lowest left ventricular internal diameter at diastole (LVIDd), left ventricular internal diameter at systole (LVIDs), and fibrosis area (reduced from ~70% to 20%) as well as highest ejection fraction (EF), fractional shortening (FS) values, and infarct thickness (increased from ~0.8 mm to 2.0 mm).

3D bioprinting brings a facile way to construct hydrogel bioelectrode arrays with geometrically complex materials and structures [70,235]. By incorporating PEDOT:PSS with the hydrogel precursor ink (mixture of PVA, chitosan, and PAA-NHS), 3D printable conducting (3DP) 3-layered hydrogel bioelectronics was fabricated (Fig. 8Bii) [9]. The outer substrate layer endows 3DP hydrogel with bioadhesion to various biological tissues (porcine skin, heart, blood vessel, and sciatic nerve) that benefits in vivo ECG monitoring. The 3DP hydrogel can provide electrical modulation for MI treatment. With a pulse stimulation (50 mV, frequency of 8 Hz, duration of 4 ms), isoproterenol-induced MI was recovered with normal synchronized pacing traces and autonomous rhythm, validating its potential application as integrated diagnosis and treatment bioelectronics.

Post-surgery real-time monitoring would provide messages and accelerate the healing process. For example, multifilament surgical sutures were reported with ability to monitor physicochemical states of deep surgical sites such as wound integrity, gastric leakage, and tissue micromotions [236]. Such monitoring of post-MI is necessary for its reusability, and it is better to be combined with on-demand therapy. A hydrogel suture (DTMS) with the capability to diagnose, treat, and monitor the infarcted heart was presented recently [237]. The DTMS is a conductive PPy deprotonated PVA microchannel hydrogel with adjustable sizes (depending on mold), large breakage strength, low friction, high conductivity (1.47 S/cm), and bidirectional perfusion function. By adding force-luminescent agent ZnS:Cu into the hydrogel precursor, the DTMS demonstrates the force-induced luminescence and photothermal conversion effect, which may provide additional bacterial clearance function. *N*-acetyl-3-nitrososulfanyl-valine (SNAP) was incorporated into DTMS via microfluidic channels as nitric oxide (NO) donor drug to construct the theranostic platform. Specifically, DTMS was used for bioelectric signal biosensor and incorporated into mobile phone by Bluetooth module (e.g., EMG and ECG) and SNAP for treatment by inhibiting inflammation and fibrosis, as well as promoting cell infiltration and vascular regeneration. The cardiac repair function of the theranostic platform was further investigated in MI minipig model (Fig. 8Biii). Characterization [cardiac magnetic resonance imaging (cMRI), immunofluorescence staining, and ECG] results confirmed that S-DTMS can promote cardiac tissue recovery, indicating the promising application of S-DTMS as the “diagnosing–treating–monitoring” closed-loop system.

#### Other electronic responsive tissues

Apart from brain and cardiac tissue, some other tissues/organs can also respond positively to electronic pulse. In a muscle injury model, the IBMN device that provides periodic in vivo ES can significantly accelerate the wound repair (>1.0 mm without ES and <0.5 mm with ES) [139]. Bone and cartilage are avascular tissues, and their self-regeneration needs external assistance for cell migration or recruitment and vascularization to supply essential oxygen and nutrient [238]. Apart from biomaterials that can provide biological cues, previous researches have proved that ES can benefit chondrogenesis and cartilage regeneration by promoting extracellular protein adsorption, facilitating cell migration or recruitment, and inducing endogenous TGF-β via the calcium signaling pathway [237,239,240]. Recently, Wang et al. [239] reported their works about the fully implantable bone defect electrical stimulation (BD-ES) system, which contains a hybrid tribo/piezoelectric nanogenerator (HTP-NG) to provide biphasic electric pulses and a conductive injectable hydrogel (EABP) to construct an osteogenic microenvironment (Fig. 8Ci). The EABP was prepared by incorporating polydopamine-modified black phosphorus nanosheets (BP@PDA) and alginate methacryloyl (AlgMA) substrate for electroactive tissue repair. The high electromechanical conversion efficiency of HTP-NG makes the BD-ES system independent of charging and circuit. The fully implantable battery-free BD-ES system would promote bone regeneration via several aspects, such as enriched signaling pathways, enhanced mechanosensitive ion channel activity, activation of calcium channel, and activation of osteogenic genes. Immunohistochemical and immunofluorescence staining confirmed that EABP in response to ES from HTP-NG could up-regulate the expression of angiogenesis/osteogenesis-related markers that promote bone repair (Fig. 8Cii).

Gastric electrical stimulation (GES) has been proved by FDA for gastroparesis under a humanitarian device exemption (HDE) via electrical impulses (0.3 ms, 14 Hz) [241]. However, implantation of GES was commonly through endoscopic placement. Ingestible electroceutical capsules may provide a noninvasive device for GES without off-target effects. Inspired by the water wicking skin of Moloch horridus, a capsule for active stimulation and hormone modulation (FLASH) was developed, which consisted of internal batteries, printed circuit boards, external electrodes, and grooved surface for fluid-wicking to remain robust electrode–mucosa contact in the presence of gastric juice [242]. It was confirmed that the FLASH was able to modulate ghrelin and glucagon-like peptide 1 (GLP-1) in plasma under ES. Furthermore, bioelectronics has also shown potential in fully artificial tissues, e.g., neuromorphic sensorimotor loop control of leg twitching was realized by artificial skin [209]. By using a metal–polymer conductor consisting of poly(l-lactide-co-ε-caprolactone) (PLC) and liquid metal [gallium–indium alloy (EGAIn)] (MPC-PLC), the biodegradable and biocompatible MPC-PLC membrane seeded with blood vessel cells [human umbilical vein endothelial cells (HUVEs), smooth muscle cells (SMCs), and fibroblasts] was rolled with a mandrel to form electronic blood vessel. After implantation in rabbit, the endothelialization process was facilitated by electrical stimulation. The diameter of electronic blood vessel remained unchanged, and it exhibited excellent patency and biosafety after implantation for 3 months [243].

## Conclusion and Perspectives

Implantable bioelectronics is facing barriers including material and function mismatch, despite that more and more flexible and stretchable materials have been incorporated to construct soft and tough bioelectronics from the aspect of electrode, substrate, encapsulation, and adhesive layers. For in vivo application, programmable stability is also important for bioelectronics apart from biocompatibility, while smart triggered biodegradable materials are rarely adopted. Bioelectronics loaded with drugs to modulate FBR or local disease of implant site has been reported, yet application of bioactive materials such as polysaccharide and protein in this area needs more profound researches. Injectable bioelectronics causes less injury during implantation; however, seamless fitting with the target tissue or organ depends mostly on shape-changing ability of bioelectronics. Living synthelectronics is emerging, which takes metabolite as driving forces for in vivo processing of soft bioelectronics. The in situ fabrication can acquire substrate-free bioelectronics while meeting the specific topological requirement of target biological substructures. To sum up, designing implantable bioelectronics shall be more closely linked with the dynamic nature of in vivo environment. The combination of biomedical materials with bioelectronics system can be further enhanced.

## Data Availability

Data sharing is not applicable to this article, as no new data were created or analyzed in this study. All data presented in this review were reused under permission.

## References

[1] Zhang T, Liu N, Xu J, Liu Z, Zhou Y, Yang Y, Li S, Huang Y, Jiang S. Flexible electronics for cardiovascular healthcare monitoring. Innovations. 2023;4(5): Article 100485. 10.1016/j.xinn.2023.100485 PMC10440597 37609559

[2] Sheng H, Zhang X, Liang J, Shao M, Xie E, Yu C, Lan W. Recent advances of energy solutions for implantable bioelectronics. Adv Healthc Mater. 2021;10(17):2100199. 10.1002/adhm.202100199 33930254

[3] Zhu S, Zhou Q, Yi J, Xu Y, Fan C, Lin C, Wu J, Lin Y. Using wool keratin as a structural biomaterial and natural mediator to fabricate biocompatible and robust bioelectronic platforms. Adv Sci. 2023;10(11):2207400. 10.1002/advs.202207400 PMC10104662 36807836

[4] Han M, Chen L, Aras K, Liang C, Chen X, Zhao H, Li K, Faye NR, Sun B, Kim JH, et al. Catheter-integrated soft multilayer electronic arrays for multiplexed sensing and actuation during cardiac surgery. Nat Biomed Eng. 2020;4(10):997–1009. 32895515 10.1038/s41551-020-00604-w PMC8021456

[5] Zhang J, Li J, Huang Z, Huang D, Yu H, Li Z. Recent progress in wearable brain–computer interface (BCI) devices based on electroencephalogram (EEG) for medical applications: A review. Health Data Sci. 2023;3:0096. 38487198 10.34133/hds.0096 PMC10880169

[6] Guimarães CF, Gasperini L, Marques AP, Reis RL. The stiffness of living tissues and its implications for tissue engineering. Nat Rev Mater. 2020;5:351–370.

[7] Akintola AA, van de Pol V, Bimmel D, Maan AC, van Heemst D. Comparative analysis of the equivital EQ02 lifemonitor with holter ambulatory ECG device for continuous measurement of ECG, heart rate, and heart rate variability: A validation study for precision and accuracy. Front Physiol. 2016;7:391. 27708585 10.3389/fphys.2016.00391 PMC5030218

[8] Hong YJ, Jeong H, Cho KW, Lu N, Kim DH. Wearable and implantable devices for cardiovascular healthcare: From monitoring to therapy based on flexible and stretchable electronics. Adv Funct Mater. 2019;29(19):1808247.

[9] Wang F, Xue Y, Chen X, Zhang P, Shan L, Duan Q, Xing J, Lan Y, Lu B, Liu J. 3D printed implantable hydrogel bioelectronics for electrophysiological monitoring and electrical modulation. Adv Funct Mater. 2023;34(21):2314471.

[10] Ye Y, Wang H, Tian Y, Gao K, Wang M, Wang X, Liang Z, You X, Gao S, Shao D, et al. Smart epidermal electrophysiological electrodes: Materials, structures, and algorithms. Nanotechnol Precis Eng. 2023;6: Article 045001.

[11] Song E, Li J, Won SM, Bai W, Rogers JA. Materials for flexible bioelectronic systems as chronic neural interfaces. Nat Mater. 2020;19(6):590–603. 32461684 10.1038/s41563-020-0679-7

[12] Xie C, Liu J, Fu TM, Dai X, Zhou W, Lieber CM. Three-dimensional macroporous nanoelectronic networks as minimally invasive brain probes. Nat Mater. 2015;14(12):1286–1292. 26436341 10.1038/nmat4427

[13] Wang W, Xu Y, Shen H. Polycrystalline silicon thin-film solar cells on various substrates. Phys Status Solidi A. 2006;203(4):721–731.

[14] Koo J, Kim SB, Choi YS, Xie Z, Bandodkar AJ, Khalifeh J, Yan Y, Kim H, Pezhouh MK, Doty K, et al. Wirelessly controlled , bioresorbable drug delivery device with active valves that exploit electrochemically triggered crevice corrosion. Sci Adv. 2020;6(35):eabb1093. 32923633 10.1126/sciadv.abb1093 PMC7455185

[15] Mariello M, Kim K, Wu K, Lacour S, Leterrier Y. Recent advances in encapsulation of flexible bioelectronic implants: Materials, technologies, and characterization methods. Adv Mater. 2022;34(34):2201129. 10.1002/adma.202201129 35353928

[16] Abyzova E, Dogadina E, Rodriguez RD, Petrov I, Kolesnikova Y, Zhou M, Liu C, Sheremet E. Beyond tissue replacement: The emerging role of smart implants in healthcare. Mater Today Bio. 2023;22: Article 100784. 10.1016/j.mtbio.2023.100784 PMC10507164 37731959

[17] Huang X, Liu L, Lin YH, Feng R, Shen Y, Chang Y, Zhao H. High-stretchability and low-hysteresis strain sensors using origami-inspired 3D mesostructures. Sci Adv. 2023;9(34):eadh9799. 37624897 10.1126/sciadv.adh9799 PMC10456843

[18] Sim K, Ershad F, Zhang Y, Yang P, Shim H, Rao Z, Lu Y, Thukral A, Elgalad A, Xi Y, et al. An epicardial bioelectronic patch made from soft rubbery materials and capable of spatiotemporal mapping of electrophysiological activity. Nat Electron. 2020;3(12):775–784.

[19] Wang S, Nie Y, Zhu H, Xu Y, Cao S, Zhang J, Li Y, Wang J, Ning X, Kong D. Intrinsically stretchable electronics with ultrahigh deformability to monitor dynamically moving organs. Sci Adv. 2022;8(13):eabl5511. 35353566 10.1126/sciadv.abl5511 PMC8967218

[20] Fallegger F, Schiavone G, Lacour SP. Conformable hybrid systems for implantable bioelectronic interfaces. Adv Mater. 2020;32(15):1903904. 10.1002/adma.201903904 31608508

[21] Yao X, Li M, He S, Jing L, Li C, Tao J, Hui X, Gao F, Song J, Chen H, et al. Kirigami-triggered spoof plasmonic interconnects for radiofrequency elastronics. Research. 2024;7:0364. 38694204 10.34133/research.0367 PMC11062506

[22] Pacchioni G. A stretchable transistor for neuromorphic devices. Nat Rev Mater. 2022;7:847.

[23] Gao Y, Chen J, Han X, Pan Y, Wang P, Wang T, Lu T. A universal strategy for tough adhesion of wet soft material. Adv Funct Mater. 2020;30(36):2003207.

[24] Han IK, Il SK, Jung SM, Jo Y, Kwon J, Chung T, Yoo S, Jang J, Kim YT, Hwang DS, et al. Electroconductive, adhesive, non-swelling, and viscoelastic hydrogels for bioelectronics. Adv Mater. 2023;35(4):e2203431. 35816086 10.1002/adma.202203431

[25] Li Z, Lu J, Ji T, Xue Y, Zhao L, Zhao K, Jia B, Wang B, Wang J, Zhang S, et al. Self-healing hydrogel bioelectronics. Adv Mater. 2024;36(21):e2306350. 37987498 10.1002/adma.202306350

[26] Zhou T, Yuk H, Hu F, Wu J, Tian F, Roh H, Shen Z, Gu G, Xu J, Lu B, et al. 3D printable high-performance conducting polymer hydrogel for all-hydrogel bioelectronic interfaces. Nat Mater. 2023;22(7):895–920. 37322141 10.1038/s41563-023-01569-2

[27] Yu Y, Guo J, Sun L, Zhang X, Zhao Y. Microfluidic generation of microsprings with ionic liquid encapsulation for flexible electronics. Research. 2019;2019:6906275. 31549079 10.34133/2019/6906275 PMC6750041

[28] Vickridge V, Ganem J, Hoshino Y, Trimaille I. Growth of SiO_2_ on SiC by dry thermal oxidation: Mechanisms. J Phys D Appl Phys. 2007;40:6254.

[29] Zhang D, Chen Q, Shi C, Chen M, Ma K, Wan J, Liu R. Dealing with the foreign-body response to implanted biomaterials: Strategies and applications of new materials. Adv Funct Mater. 2021;31(6):2007226.

[30] Lee S, Park S, Park J, Lee JY. Implantable polypyrrole bioelectrodes inducing anti-inflammatory macrophage polarization for long-term in vivo signal recording. Acta Biomater. 2023;168:458–469. 37414115 10.1016/j.actbio.2023.06.042

[31] Li Y, Li N, De Oliveira O, Wang S. Implantable bioelectronics toward long-term stability and sustainability. Matter. 2021;4(4):1125–1141.

[32] Choi YS, Koo J, Lee YJ, Lee G, Avila R, Ying H, Reeder J, Hambitzer L, Im K, Kim J, et al. Biodegradable polyanhydrides as encapsulation layers for transient electronics. Adv Funct Mater. 2020;30(31):2000941.

[33] Wu M, Yao K, Huang N, Li H, Zhou J, Shi R, Li J, Huang X, Li J, Jia H, et al. Ultrathin, soft, bioresorbable organic electrochemical transistors for transient spatiotemporal mapping of brain activity. Adv Sci. 2023;10(14):2300504. 10.1002/advs.202300504 PMC10190644 36825679

[34] Liu S, Jia Z, Yang F, Ning T, Gu X, Niu X, Fan Y. Flexible transient bioelectronic system enables multifunctional active-controlled drug delivery. Adv Funct Mater. 2023;33(13):2215034.

[35] Zhong S, Yao S, Zhao Q, Wang Z, Liu Z, Li L, Wang ZL. Electricity-assisted cancer therapy: From traditional clinic applications to emerging methods integrated with nanotechnologies. Adv Nanobiomed Res. 2023;3(3):2200143.

[36] Wang H, Li S, Lu H, Zhu M, Liang H, Wu X, Zhang Y. Carbon-based flexible devices for comprehensive health monitoring. Small Methods. 2023;7:e2201340. 36617527 10.1002/smtd.202201340

[37] Jiang Y, Trotsyuk AA, Niu S, Henn D, Chen K, Shih C, Larson MR, Mermin-Bunnell AM, Mittal S, Lai JC, et al. Wireless, closed-loop, smart bandage with integrated sensors and stimulators for advanced wound care and accelerated healing. Nat Biotechnol. 2023;41(5):652–662. 36424488 10.1038/s41587-022-01528-3

[38] Yuk H, Wu J, Zhao X. Hydrogel interfaces for merging humans and machines. Nat Rev Mater. 2022;7(12):935–952.

[39] Kang T, Cha GD, Park OK, Cho HR, Kim M, Lee J, Kim D, Lee B, Chu J, Koo S, et al. Penetrative and sustained drug delivery using injectable hydrogel nanocomposites for postsurgical brain tumor treatment. ACS Nano. 2023;17(6):5435–5447. 36926815 10.1021/acsnano.2c10094

[40] Jiang X, Wu H, Xiao A, Huang Y, Yu X, Chang L. Recent advances in bioelectronics for localized drug delivery. Small Methods. 2024;8(1):2301068. 10.1002/smtd.202301068 37759393

[41] Liu J, Lin S, Li W, Zhao Y, Liu D, He Z, Wang D, Lei M, Hong B, Wu H. Ten-hour stable noninvasive brain-computer interface realized by semidry hydrogel-based electrodes. Research. 2022;2022:9830457. 35356767 10.34133/2022/9830457 PMC8933689

[42] Zhao Q, Zhu M, Tian G, Liang C, Liu Z, Huang J, Yu QY, Tang S, Chen J, Zhao X, et al. Highly sensitive and omnidirectionally stretchable bioelectrode arrays for in vivo neural interfacing. Adv Healthc Mater. 2023;12(18):e2203344. 36974567 10.1002/adhm.202203344

[43] Kang M, Park J, Kim SA, Young T, Yeon J, Woo D, Park K, Seo J. Modulus-tunable multifunctional hydrogel ink with nanofillers for 3D-printed soft electronics. Biosens Bioelectron. 2024;255: Article 116257. 38574560 10.1016/j.bios.2024.116257

[44] Park J, Kim JY, Heo JH, Kim Y, Kim SA, Park K, Lee Y, Jin Y, Shin SR, Kim DW, et al. Intrinsically nonswellable multifunctional hydrogel with dynamic nanoconfinement networks for robust tissue-adaptable bioelectronics. Adv Sci. 2023;10(12):2207237. 10.1002/advs.202207237 PMC10131858 36799540

[45] Gablech I, Głowacki ED. State-of-the-art electronic materials for thin films in bioelectronics. Adv Electron Mater. 2023;9(8):2300258.

[46] Balakrishnan G, Song J, Mou C, Bettinger CJ. Recent progress in materials chemistry to advance flexible bioelectronics in medicine. Adv Mater. 2022;34(10):e2106787. 34751987 10.1002/adma.202106787 PMC8917047

[47] Sunwoo SH, Han SI, Kang H, Cho YS, Jung D, Lim C, Lim C, Cha MJ, Lee SP, Hyeon T, et al. Stretchable low-impedance nanocomposite comprised of Ag–Au core–shell nanowires and Pt black for epicardial recording and stimulation. Adv Mater Technol. 2020;5(3):1900768.

[48] Park B, Shin JH, Ok J, Park S, Jung W, Jeong C, Choy S, Jo YJ, Kim TI. Cuticular pad–inspired selective frequency damper for nearly dynamic noise–free bioelectronics. Science. 2022;376(6593):624–629. 35511972 10.1126/science.abj9912

[49] Shihada JA, Jung M, Decke S, Koschinski L, Musall S, Montes VR, Offenhäusser A. Highly customizable 3D microelectrode arrays for in vitro and in vivo neuronal tissue recordings. Adv Sci. 2024;11(13):e2305944. 10.1002/advs.202305944 PMC10987114 38240370

[50] Coughlin B, Muñoz W, Kfir Y, Young MJ, Meszéna D, Jamali M, Caprara I, Hardstone R, Khanna A, Mustroph ML, et al. Modified Neuropixels probes for recording human neurophysiology in the operating room. Nat Protoc. 2023;18(10):2927–2953. 37697108 10.1038/s41596-023-00871-2

[51] Ju J, Feleke AG, Luo L, Fan X. Recognition of drivers’ hard and soft braking intentions based on hybrid brain-computer interfaces. Cyborg Bionic Syst. 2022;2022:9847652. 39886316 10.34133/2022/9847652 PMC11781701

[52] Hu M, Liang C, Wang D. Implantable bioelectrodes: Challenges, strategies, and future directions. Biomater Sci. 2024;12(2):270–287. 38175154 10.1039/d3bm01204b

[53] Lin Z, Kireev D, Liu N, Gupta S, LaPiano J, Obaid SN, Chen Z, Akinwande D, Efimov IR. Graphene biointerface for cardiac arrhythmia diagnosis and treatment. Adv Mater. 2023;35(22):e2212190. 36965107 10.1002/adma.202212190 PMC12013714

[54] Driscoll N, Erickson B, Murphy BB, Richardson AG, Robbins G, Apollo NV, Mentzelopoulos G, Mathis T, Hantanasirisakul K, Bagga P, et al. MXene-infused bioelectronic interfaces for multiscale electrophysiology and stimulation. Sci Transl Med. 2021;13(612):eabf8629. 34550728 10.1126/scitranslmed.abf8629 PMC8722432

[55] Jiang Y, Zhang Z, Wang Y, Li D, Coen CT, Hwaun E, Chen G, Wu HC, Zhong D, Niu S, et al. Topological supramolecular network enabled high-conductivity, stretchable organic bioelectronics. Science. 2022;375(6587):1411–1417. 35324282 10.1126/science.abj7564

[56] Qi D, Liu Z, Liu Y, Jiang Y, Leow WR, Pal M, Pan S, Yang H, Wang Y, Zhang X, et al. Highly stretchable, compliant, polymeric microelectrode arrays for in vivo electrophysiological interfacing. Adv Mater. 2017;29(40):1702800. 10.1002/adma.201702800 28869690

[57] Wu J, Li Y, Duan S, Wang Z, Jing X, Lin Y, Zhu D, Lei W, Shi Q, Tao L. Bioinspired stretchable MXene deformation-insensitive hydrogel temperature sensors for plant and skin electronics. Research. 2023;6:0106. 37275122 10.34133/research.0106 PMC10237174

[58] Ji B, Wang M. Micro-wrinkle strategy for stable soft neural interface with optimized electroplated PEDOT: PSS. J Micromech Microeng. 2022;30: Article 104001.

[59] Driscoll N, Erickson B, Murphy BB, Richardson AG, Robbins G, Apollo NV, Mentzelopoulos G, Mathis T, Hantanasirisakul K, Bagga P, et al. MXene-infused bioelectronic interfaces for multiscale electrophysiology and stimulation. Sci Transl Med. 2021;13(612):eabf8629. 34550728 10.1126/scitranslmed.abf8629 PMC8722432

[60] Lee M, Park J, Choe G, Lee S, Kang BG, Jun JH, Shin Y, Kim MC, Kim YS, Ahn Y, et al. A conductive and adhesive hydrogel composed of MXene nanoflakes as a paintable cardiac patch for infarcted heart repair. ACS Nano. 2023;17(13):12290–12304. 37339066 10.1021/acsnano.3c00933

[61] Cho YU, Kim K, Dutta A, Park SH, Lee JY, Kim HW, Park J, Kim J, Min WK, Won C, et al. MRI-compatible, transparent pedot:PSS neural implants for the alleviation of neuropathic pain with motor cortex stimulation. Adv Funct Mater. 2024;34(6):2310908.

[62] Yi Z, Zhan F, Chen Y, Zhang R, Lin H, Zhao L. An electroconductive hydrogel with injectable and self-healing properties accelerates peripheral nerve regeneration and motor functional recovery. Chem Eng J. 2023;478:147261.

[63] Li X, Tan Z, Guo B, Yu C, Yao M, Liang L, Wu X, Zhao Z, Yao F, Zhang H, et al. Magnet-oriented hydrogels with mechanical–electrical anisotropy and photothermal antibacterial properties for wound repair and monitoring. Chem Eng J. 2023;463:142387.

[64] Bin YW, Wang G, Kong Z, Yao CK, Wang Y, Hu H, Li F, Chen C, Tian Y, Zhang J, et al. A biologically muscle-inspired polyurethane with super-tough, thermal reparable and self-healing capabilities for stretchable electronics. Adv Funct Mater. 2021;31(10):2009869.

[65] Chen Z, Zhang T, Chen CT, Yang S, Lv Z, Cao L, Ren J, Shao Z, Jiang LB, Ling S. Mechanically and electrically biocompatible hydrogel ionotronic fibers for fabricating structurally stable implants and enabling noncontact physioelectrical modulation. Mater Horiz. 2022;9(6):1735–1749. 35502878 10.1039/d2mh00296e

[66] Hui Y, Yao Y, Qian Q, Luo J, Chen H, Qiao Z, Yu Y, Tao L, Zhou N. Three-dimensional printing of soft hydrogel electronics. Nat Electron. 2022;5(12):893–903.

[67] Chong J, Sung C, Nam KS, Kang T, Kim H, Lee H, Park H, Park S, Kang J. Highly conductive tissue-like hydrogel interface through template-directed assembly. Nat Commun. 2023;14(1):2206. 37072411 10.1038/s41467-023-37948-1 PMC10113367

[68] Li GL, Wu JT, Xia YH, He QG, Jin HG. Review of semi-dry electrodes for EEG recording. J Neural Eng. 2020;17(5): Article 051004. 33002886 10.1088/1741-2552/abbd50

[69] Li G, Wang S, Li M, Duan YY. Towards real-life EEG applications: Novel superporous hydrogel-based semi-dry EEG electrodes enabling automatically “charge-discharge” electrolyte. J Neural Eng. 2021;18(4): Article 046016. 10.1088/1741-2552/abeeab 33721854

[70] Li G, Liu Y, Chen Y, Xia Y, Qi X, Wan X, Jin Y, Liu J, He Q, Li K, et al. Robust, self-adhesive, and low-contact impedance polyvinyl alcohol/polyacrylamide dual-network hydrogel semidry electrode for biopotential signal acquisition. SmartMat. 2024;5(2): Article e1173.

[71] Chen Y, Chang Z, Liu Y, Wan X, Wang T, Zhou Z, Li G. Tongue-inspired gelatin/poly(acrylate-co-acrylamide)-Fe^3+^ organic hydrogel with tunable mechanical, electrical, and sensory properties. Eur Polym J. 2024;210: Article 112992.

[72] Chen Z, Lin Z, Obaid SN, Rytkin E, George SA, Bach C, Madrid M, Liu M, LaPiano J, Fehr A, et al. Soft, bioresorbable, transparent microelectrode arrays for multimodal spatiotemporal mapping and modulation of cardiac physiology. Sci Adv. 2023;9(27):eadi0757. 37406128 10.1126/sciadv.adi0757 PMC10321742

[73] Hu Z, Guo H, An D, Wu M, Kaura A, Oh H, Wang Y, Zhao M, Li S, Yang Q, et al. Bioresorbable multilayer organic—Inorganic films for bioelectronic systems. Adv Mater. 2024;36(19):e2309421. 38339983 10.1002/adma.202309421

[74] Xue Y, Chen X, Wang F, Lin J, Liu J. Mechanically-compliant bioelectronic interfaces through fatigue-resistant conducting polymer hydrogel coating. Adv Mater. 2023;35(40):e2304095. 37381603 10.1002/adma.202304095

[75] Sani ES, Xu C, Wang C, Song Y, Min J, Tu J, Solomon SA, Li J, Banks JL, Armstrong DG, et al. A stretchable wireless wearable bioelectronic system for multiplexed monitoring and combination treatment of infected chronic wounds. Sci Adv. 2023;9(12):eadf7388. 36961905 10.1126/sciadv.adf7388 PMC10038347

[76] Cheng P, Dai S, Liu Y, Li Y, Hayashi H, Papani R, Su Q, Li N, Dai Y, Liu W, et al. An intrinsically stretchable power-source system for bioelectronics. Dev Dent. 2024;2(1): Article 100216.

[77] Zhuang Q, Yao K, Wu M, Lei Z, Chen F, Li J, Mei Q, Zhou Y, Huang Q, Zhao X, et al. Wafer-patterned, permeable, and stretchable liquid metal microelectrodes for implantable bioelectronics with chronic biocompatibility. Sci Adv. 2023;9(22):eadg8602. 37256954 10.1126/sciadv.adg8602 PMC10413659

[78] Li N, Li Y, Cheng Z, Liu Y, Dai Y, Kang S, Li S, Shan N, Wai S, Ziaja A, et al. Bioadhesive polymer semiconductors and transistors for intimate biointerfaces. Science. 2023;693(6658):686–693. 10.1126/science.adg8758 PMC10768720 37561870

[79] Hu Z, Zhao J, Guo H, Li R, Wu M, Shen J, Wang Y, Qiao Z, Xu Y, Haugstad G, et al. Ultrathin,transferred layers of silicon oxynitrides as tunable biofluid barriers for bioresorbable electronic systems. Adv Mater. 2024;36(15):e2307782. 38303684 10.1002/adma.202307782

[80] Strakosas X, Biesmans H, Abrahamsson T, Hellman K, Ejneby MS, Donahue MJ, Ekström P, Ek F, Savvakis M, Hjort M, et al. Metabolite-induced in vivo fabrication of substrate-free organic bioelectronics. Science. 2023;379(6634):795–802. 36821679 10.1126/science.adc9998

[81] Moon H, Jang JW, Park S, Kim JH, Kim JS, Kim S. Soft, conformal PDMS-based ECoG electrode array for long-term in vivo applications. Sensors Actuators B Chem. 2024;401: Article 135099.

[82] Song E, Chiang C, Li R, Jin X, Zhao J, Hill M, Xia Y, Li L, Huang Y, Won SM, et al. Flexible electronic/optoelectronic microsystems with scalable designs for chronic biointegration. Proc Natl Acad Sci USA. 2019;116(31):15398–15406. 31308234 10.1073/pnas.1907697116 PMC6681732

[83] Li H, Liu H, Sun M, Huang YA, Xu L. 3D interfacing between soft electronic tools and complex biological tissues. Adv Mater. 2021;33(3):2004425. 10.1002/adma.202004425 33283351

[84] Qu C, Guo Q, Wu X, You C, Wu B, Zhang Z, Mei Y. Matryoshka-inspired continuous assembly of flexible silicon microribbons and photodetectors via selective transfer printing. Mater Today Phys. 2023;35: Article 101090.

[85] Yi H, Seong M, Sun K, Hwang I, Lee K, Cha C, Kim TI, Jeong HE. Wet-responsive, reconfigurable, and biocompatible hydrogel adhesive films for transfer printing of nanomembranes. Adv Funct Mater. 2018;28(18):1706498.

[86] Cao J, Liu X, Qiu J, Yue Z, Li Y, Xu Q, Chen Y, Chen J, Cheng H, Xing G, et al. Anti-friction gold-based stretchable electronics enabled by interfacial diffusion-induced cohesion. Nat Commun. 2024;15(1):1116. 38321072 10.1038/s41467-024-45393-x PMC10847152

[87] Ji B, Sun F, Guo J, Zhou Y, You X, Fan Y, Wang L, Xu M, Zeng W, Liu J, et al. Brainmask: An ultrasoft and moist micro-electrocorticography electrode for accurate positioning and long-lasting recordings. Microsyst Nanoeng. 2023;9:126. 37829160 10.1038/s41378-023-00597-x PMC10564857

[88] Seo H, Hong YM, Chung WG, Park W, Lee J, Kim HK, Byeon SH, Kim DW, Park JU. Real- time in vivo monitoring of intraocular pressure distribution in the anterior chamber and vitreous chamber for diagnosis of glaucoma. Sci Adv. 2024;10(6):eadk7805. 38324695 10.1126/sciadv.adk7805 PMC10851251

[89] Rao Z, Lu Y, Li Z, Sim K, Ma Z, Xiao J, Yu C. Curvy, shape-adaptive imagers based on printed optoelectronic pixels with a kirigami design. Nat Electron. 2021;4(7):513–521.

[90] Shim H, Ershad F, Patel S, Zhang Y, Wang B, Chen Z, Marks TJ, Facchetti A, Yu C. An elastic and reconfigurable synaptic transistor based on a stretchable bilayer semiconductor. Nat Electron. 2022;5:660–671.

[91] Orts Mercadillo V, Chan KC, Caironi M, Athanassiou A, Kinloch IA, Bissett M, Cataldi P. Electrically conductive 2D material coatings for flexible and stretchable electronics: A comparative review of graphenes and MXenes. Adv Funct Mater. 2022;32(38):2204772.

[92] Wu SJ, Wu J, Kaser SJ, Roh H, Shiferaw RD, Yuk H, Zhao X. A 3D printable tissue adhesive. Nat Commun. 2024;15(1):1215. 38331971 10.1038/s41467-024-45147-9 PMC10853267

[93] Liang S, Zhang Y, Wang H, Xu Z, Chen J, Bao R, Tan B, Cui Y, Fan G, Wang W, et al. Paintable and rapidly bondable conductive hydrogels as therapeutic cardiac patches. Adv Mater. 2018;30(23):1704235. 10.1002/adma.201704235 29687502

[94] Shen S, Zhang J, Han Y, Pu C, Duan Q, Huang J, Yan B, You X, Lin R, Shen X, et al. A core–shell nanoreinforced ion-conductive implantable hydrogel bioelectronic patch with high sensitivity and bioactivity for real-time synchronous heart monitoring and repairing. Adv Healthc Mater. 2023;12(29):2301990. 10.1002/adhm.202301990 37467758

[95] Bertsch P, Diba M, Mooney DJ, Leeuwenburgh SCG. Self-healing injectable hydrogels for tissue regeneration. Chem Rev. 2023;123(2):834–873. 35930422 10.1021/acs.chemrev.2c00179 PMC9881015

[96] Seo H, Han SI, Il SK, Seong D, Lee K, Kim SH, Park T, Koo JH, Shin M, Baac HW, et al. Durable and fatigue-resistant soft peripheral neuroprosthetics for in vivo bidirectional signaling. Adv Mater. 2021;33(20):2007346. 10.1002/adma.202007346 33739558

[97] Li S, Zhang H, Xie J, Wang Z, Wang K, Zhai Z, Ding J, Wang S, Shen L, Wen J, et al. In vivo self-assembled shape-memory polyurethane for minimally invasive delivery and therapy. Mater Horiz. 2023;10(9):3438–3449. 37424353 10.1039/d3mh00594a

[98] Mou C, Wang X, Teng J, Xie Z, Zheng M. Injectable self-healing hydrogel fabricated from antibacterial carbon dots and ɛ-polylysine for promoting bacteria-infected wound healing. J Nanobiotechnol. 2022;20(1):368. 10.1186/s12951-022-01572-w PMC9367091 35953858

[99] Jin S, Choi H, Seong D, You CL, Kang JS, Rho S, Lee WB, Son D, Shin M. Injectable tissue prosthesis for instantaneous closed-loop rehabilitation. Nature. 2023;623(7985):58–65. 37914945 10.1038/s41586-023-06628-x

[100] Gai Y, Yin Y, Guan L, Zhang S, Chen J, Yang J, Zhou H, Li J. Rational design of bioactive materials for bone hemostasis and defect repair. Cyborg Bionic Syst. 2023;4:0058. 37829507 10.34133/cbsystems.0058 PMC10566342

[101] Cai C, Zhang X, Li Y, Liu X, Wang S, Lu M, Yan X, Deng L, Liu S, Wang F, et al. Self-healing hydrogel embodied with macrophage-regulation and responsive-gene-silencing properties for synergistic prevention of peritendinous adhesion. Adv Mater. 2022;34(5):2106564. 10.1002/adma.202106564 34816470

[102] Kang Y, Zhang H, Chen L, Dong J, Yao B, Yuan X, Qin D, Yaremenko AV, Liu C, Feng C, et al. The marriage of Xenes and hydrogels: Fundamentals, applications, and outlook. Innovations. 2022;3(6): Article 100327. 10.1016/j.xinn.2022.100327 PMC9573930 36263399

[103] Ren H, Zhang Z, Cheng X, Zou Z, Chen X, He C. Injectable, self-healing hydrogel adhesives with firm tissue adhesion and on-demand biodegradation for sutureless wound closure. Sci Adv. 2023;9(33):eadh4327. 37585520 10.1126/sciadv.adh4327 PMC10431709

[104] Xu J, Chen TY, Tai CH, Hsu SH. Bioactive self-healing hydrogel based on tannic acid modified gold nano-crosslinker as an injectable brain implant for treating Parkinson’s disease. Biomater Res. 2023;27(1):8. 36755333 10.1186/s40824-023-00347-0 PMC9909866

[105] Kang J, Son D, Wang GJN, Liu Y, Lopez J, Kim Y, Oh JY, Katsumata T, Mun J, Lee Y, et al. Tough and water-insensitive self-healing elastomer for robust electronic skin. Adv Mater. 2018;30(13):1706846. 10.1002/adma.201706846 29424026

[106] Yu H, Chen C, Sun J, Zhang H, Feng Y, Qin M, Feng W. Highly thermally conductive polymer/graphene composites with rapid room-temperature self-healing capacity. Nanomicro Lett. 2022;14(1):135. 35704244 10.1007/s40820-022-00882-w PMC9200911

[107] Jiang C, Zhang L, Yang Q, Huang S, Shi H, Long Q, Qian B, Liu Z, Guan Q, Liu M, et al. Self-healing polyurethane-elastomer with mechanical tunability for multiple biomedical applications in vivo. Nat Commun. 2021;12(1):4395. 34285224 10.1038/s41467-021-24680-x PMC8292539

[108] Zhou L, Zhang L, Li P, Maitz MF, Wang K, Shang T, Dai S, Fu Y, Zhao Y, Yang Z, et al. Adhesive and self-healing polyurethanes with tunable multifunctionality. Research. 2022;2022:9795682. 36349335 10.34133/2022/9795682 PMC9639449

[109] Son D, Kang J, Vardoulis O, Kim Y, Matsuhisa N, Oh JY, To JWF, Mun J, Katsumata T, Liu Y, et al. An integrated self-healable electronic skin system fabricated via dynamic reconstruction of a nanostructured conducting network. Nat Nanotechnol. 2018;13(11):1057–1065. 30127474 10.1038/s41565-018-0244-6

[110] Cooper CB, Root SE, Michalek L, Wu S, Lai JC, Khatib M, Oyakhire ST, Zhao R, Qin J, Bao Z. Autonomous alignment and healing in multilayer soft electronics using immiscible dynamic polymers. Science. 2023;380(6648):935–941. 37262169 10.1126/science.adh0619

[111] Oh JY, Rondeau-Gagné S, Chiu YC, Chortos A, Lissel F, Wang GJN, Schroeder BC, Kurosawa T, Lopez J, Katsumata T, et al. Intrinsically stretchable and healable semiconducting polymer for organic transistors. Nature. 2016;539(7629):411–415. 27853213 10.1038/nature20102

[112] Sun C, Cheng Z, Abu-Halimah J, Tian B. Perspectives on tissue-like bioelectronics for neural modulation. iScience. 2023;26(5): Article 106715. 37216128 10.1016/j.isci.2023.106715 PMC10192532

[113] Li X, Jiang H, He N, Yuan WE, Qian Y, Ouyang Y. Graphdiyne-related materials in biomedical applications and their potential in peripheral nerve tissue engineering. Cyborg Bionic Syst. 2022;2022:9892526. 36285317 10.34133/2022/9892526 PMC9494693

[114] Park S, Yuk H, Zhao R, Yim YS, Woldeghebriel EW, Kang J, Canales A, Fink Y, Choi GB, Zhao X, et al. Adaptive and multifunctional hydrogel hybrid probes for long-term sensing and modulation of neural activity. Nat Commun. 2021;12(1):3435. 34103511 10.1038/s41467-021-23802-9 PMC8187649

[115] Axpe E, Orive G, Franze K, Appel EA. Towards brain-tissue-like biomaterials. Nat Commun. 2020;11(1):3423. 32647269 10.1038/s41467-020-17245-x PMC7347841

[116] Mredha MTI, Le HH, Tran VT, Trtik P, Cui J, Jeon I. Anisotropic tough multilayer hydrogels with programmable orientation. Mater Horiz. 2019;6(7):1504–1511.

[117] Sun Q, Ma S, Lin P, Wang X, Zheng Z, Zhou F. Anisotropic hydrogels with high mechanical strength by stretching-induced oriented crystallization and drying. ACS Appl Polym Mater. 2020;2(6):2142–2150.

[118] Lang C, Lloyd EC, Matuszewski KE, Xu Y, Ganesan V, Huang R, Kumar M, Hickey RJ. Nanostructured block copolymer muscles. Nat Nanotechnol. 2022;17(7):752–758. 35654867 10.1038/s41565-022-01133-0

[119] Kim IH, Choi S, Lee J, Jung J, Yeo J, Kim JT, Ryu S, Ahn SK, Kang J, Poulin P, et al. Human-muscle-inspired single fibre actuator with reversible percolation. Nat Nanotechnol. 2022;17(11):1198–1205. 36302962 10.1038/s41565-022-01220-2 PMC9646516

[120] Kanik M, Orguc S, Varnavides G, Kim J, Benavides T, Gonzalez D, Akintilo T, Tasan CC, Chandrakasan AP, Fink Y, et al. Strain-programmable fiber-based artificial muscle. Science. 2019;365(6449):145–150. 31296764 10.1126/science.aaw2502 PMC7262675

[121] Aldana AA, Valente F, Dilley R, Doyle B. Development of 3D bioprinted GelMA-alginate hydrogels with tunable mechanical properties. Bioprinting. 2021, 21: Article e00105.

[122] Pan L, Wang F, Cheng Y, Leow WR, Zhang YW, Wang M, Cai P, Ji B, Li D, Chen X. A supertough electro-tendon based on spider silk composites. Nat Commun. 2020;11(1):1332. 32165612 10.1038/s41467-020-14988-5 PMC7067870

[123] Park J, Lee S, Lee M, Kim HS, Lee JY. Injectable conductive hydrogels with tunable degradability as novel implantable bioelectrodes. Small. 2023;19(21):2300250. 10.1002/smll.202300250 36828790

[124] Torculas M, Medina J, Xue W, Hu X. Protein-based bioelectronics. ACS Biomater Sci Eng. 2016;2(8):1211–1223. 33465848 10.1021/acsbiomaterials.6b00119

[125] Zhang Y, Dong L, Liu L, Wu Z, Pan D, Liu L. Recent advances of stimuli-responsive polysaccharide hydrogels in delivery systems: A review. J Agric Food Chem. 2022;70(21):6300–6316. 35578738 10.1021/acs.jafc.2c01080

[126] Wang S, Zhao Q, Li J, Du X. Morphing-to-adhesion polysaccharide hydrogel for adaptive biointerfaces. ACS Appl Mater Interfaces. 2022;14(37):42420–42429. 36083279 10.1021/acsami.2c10117

[127] Yao X, Song Y, Jiang L. Applications of bio-inspired special wettable surfaces. Adv Mater. 2011;23(6):719–734. 21287632 10.1002/adma.201002689

[128] Zhang P, Lin L, Zang D, Guo X, Liu M. Designing bioinspired anti-biofouling surfaces based on a superwettability strategy. Small. 2017;13(4):1503334. 10.1002/smll.201503334 26917251

[129] Wang T, Deng J, Ran R, Shi W, Gao Y, Ren X, Cao J, Zhang M. In-situ forming PEG-engineering hydrogels with anti-fouling characteristics as an artificial vitreous body. Chem Eng J. 2022;449: Article 137486.

[130] Li Q, Wen C, Yang J, Zhou X, Zhu Y, Zheng J, Cheng G, Bai J, Xu T, Ji J, et al. Zwitterionic biomaterials. Chem Rev. 2022;122:17073–17154. 36201481 10.1021/acs.chemrev.2c00344

[131] He Z, Lan X, Hu Q, Li H, Li L, Mao J. Antifouling strategies based on super-phobic polymer materials. Prog Org Coat. 2021;157: Article 106285.

[132] Fayzullin A, Bakulina A, Mikaelyan K, Shekhter A, Guller A. Implantable drug delivery systems and foreign body reaction: Traversing the current clinical landscape. Bioengineering. 2021;8(12):205. 34940358 10.3390/bioengineering8120205 PMC8698517

[133] Klopfleisch R, Jung F. The pathology of the foreign body reaction against biomaterials. J Biomed Mater Res A. 2017;105(3):927–940. 27813288 10.1002/jbm.a.35958

[134] Chen S, Saeed AFUH, Liu Q, Jiang Q, Xu H, Xiao GG, Rao L, Duo Y. Macrophages in immunoregulation and therapeutics. Signal Transduct Target Ther. 2023;8(1):207. 37211559 10.1038/s41392-023-01452-1 PMC10200802

[135] Furman D, Campisi J, Verdin E, Carrera-Bastos P, Targ S, Franceschi C, Ferrucci L, Gilroy DW, Fasano A, Miller GW, et al. Chronic inflammation in the etiology of disease across the life span. Nat Med. 2019;25(12):1822–1832. 31806905 10.1038/s41591-019-0675-0 PMC7147972

[136] Miron RJ, Zohdi H, Fujioka-Kobayashi M, Bosshardt DD. Giant cells around bone biomaterials: Osteoclasts or multi-nucleated giant cells? Acta Biomater. 2016;46:15–28. 27667014 10.1016/j.actbio.2016.09.029

[137] Laumont CM, Banville AC, Gilardi M, Hollern DP, Nelson BH. Tumour-infiltrating B cells: Immunological mechanisms, clinical impact and therapeutic opportunities. Nat Rev Cancer. 2022;22(7):414–430. 35393541 10.1038/s41568-022-00466-1 PMC9678336

[138] Yang Q, Hu Z, Rogers JA. Functional hydrogel interface materials for advanced bioelectronic devices. Acc Mater Res. 2021;2(11):1010–1023.

[139] Fidanovski K, Mawad D. Conjugated polymers in bioelectronics: Addressing the interface challenge. Adv Healthc Mater. 2019;8(10):1900053. 10.1002/adhm.201900053 30941922

[140] Xie C, Wang X, He H, Ding Y, Lu X. Mussel-inspired hydrogels for self-adhesive bioelectronics. Adv Funct Mater. 2020;30(25):1909954.

[141] Zhang C, Wu B, Zhou Y, Zhou F, Liu W, Wang Z. Mussel-inspired hydrogels : From design principles. Chem Soc Rev. 2020;49(11):3605–3637. 32393930 10.1039/c9cs00849g

[142] Li Z, Cao H, Xu Y, Li X, Han XW, Fan Y, Jiang Q, Sun Y, Zhang X. Bioinspired polysaccharide hybrid hydrogel promoted recruitment and chondrogenic differentiation of bone marrow mesenchymal stem cells. Carbohydr Polym. 2021;267: Article 118224. 34119177 10.1016/j.carbpol.2021.118224

[143] Yao M, Sun H, Guo Z, Sun X, Yu Q, Wu X, Yu C, Zhang H, Yao F, Li J. A starch-based zwitterionic hydrogel coating for blood-contacting devices with durability and bio-functionality. Chem Eng J. 2021;421(Pt 1): Article 129702.

[144] Yan L, Zhou T, Ni R, Jia Z, Jiang Y, Guo T, Wang K, Chen X, Han L, Lu X. Adhesive gelatin-catechol complex reinforced poly(acrylic acid) hydrogel with enhanced toughness and cell affinity for cartilage regeneration. ACS Appl Bio Mater. 2022;5(9):4366–4377. 10.1021/acsabm.2c00533 36044775

[145] Wang X, Sun X, Gan D, Soubrier M, Chiang HY, Yan L, Li Y, Li J, Yu S, Xia Y, et al. Bioadhesive and conductive hydrogel-integrated brain-machine interfaces for conformal and immune-evasive contact with brain tissue. Matter. 2022;5(4):1204–1223.

[146] Hou Y, Li Y, Li Y, Li D, Guo T, Deng X, Zhang H, Xie C, Lu X. Tuning water-resistant networks in mussel-inspired hydrogels for robust wet tissue and bioelectronic adhesion. ACS Nano. 2023;17(3):2745–2760. 36734875 10.1021/acsnano.2c11053

[147] Yu C, Shi M, He S, Yao M, Sun H, Yue Z, Qiu Y, Liu B, Liang L, Zhao Z, et al. Chronological adhesive cardiac patch for synchronous mechanophysiological monitoring and electrocoupling therapy. Nat Commun. 2023;14(1):6226. 37803005 10.1038/s41467-023-42008-9 PMC10558550

[148] Lin J, Chen X, Zhang P, Xue Y, Feng Y, Ni Z, Tao Y, Wang Y, Liu J. Wireless bioelectronics for in vivo pressure monitoring with mechanically-compliant hydrogel biointerfaces. Adv Mater. 2024;36(26):2400181. 10.1002/adma.202400181 38419474

[149] Yang J, Bai R, Chen B, Suo Z. Hydrogel adhesion: A supramolecular synergy of chemistry , topology, and mechanics. Adv Funct Mater. 2020;30(2):1901693.

[150] Yang J, Bai R, Suo Z. Topological adhesion of wet materials. Adv Mater. 2018;30(25):1800671. 10.1002/adma.201800671 29726051

[151] Chen Y, Meng J, Gu Z, Wan X, Jiang L, Wang S. Bioinspired multiscale wet adhesive surfaces: Structures and controlled adhesion. Adv Funct Mater. 2020;30(5):1905287.

[152] Steck J, Kim J, Yang J, Hassan S, Suo Z. Topological adhesion. I. Rapid and strong topohesives. Extreme Mech Lett. 2020;39:100803.

[153] Shi Z, Zheng F, Zhou Z, Li M, Fan Z, Ye H, Zhang S, Xiao T, Chen L, Tao TH, et al. Silk-enabled conformal multifunctional bioelectronics for investigation of spatiotemporal epileptiform activities and multimodal neural encoding/decoding. Adv Sci. 2019;6(9):1801617. 10.1002/advs.201801617 PMC6498121 31065516

[154] Liu R, Zhao Z, Yang Q, Chen S, Yan Z, Li X, Liang L, Guo B, Wang B, Zhang H, et al. A single-component Janus zwitterionic hydrogel patch with a bionic microstructure for postoperative adhesion prevention. ACS Appl Mater Interfaces. 2024;16(18):22900–22913. 10.1021/acsami.4c01845 38669466

[155] Muir VG, Burdick JA. Chemically modified biopolymers for the formation of biomedical hydrogels. Chem Rev. 2021;121(18):10908–10949. 33356174 10.1021/acs.chemrev.0c00923 PMC8943712

[156] Li Z, Lin Z. Recent advances in polysaccharide-based hydrogels for synthesis and applications. Aggregate. 2021;2(2): Article e21.

[157] Cea C, Zhao Z, Wisniewski DJ, Spyropoulos GD, Polyvaras A, Gelinas JN, Khodagholy D. Integrated internal ion-gated organic electrochemical transistors for stand-alone conformable bioelectronics. Nat Mater. 2023;22:1227–1235. 37429941 10.1038/s41563-023-01599-w PMC10533388

[158] Lim ZX, Cheong KY. Nonvolatile memory device based on bipolar and unipolar resistive switching in bio-organic aloe polysaccharides thin film. Adv Mater Technol. 2018;3(5):1800007.

[159] Zhong C, Deng Y, Roudsari AF, Kapetanovic A, Anantram MP, Rolandi M. A polysaccharide bioprotonic field-effect transistor. Nat Commun. 2011;2:476. 21934660 10.1038/ncomms1489

[160] Tian G, Yang D, Liang C, Liu Y, Chen J, Zhao Q, Tang S, Huang J, Xu P, Liu Z, et al. A nonswelling hydrogel with regenerable high wet tissue adhesion for bioelectronics. Adv Mater. 2023;35(18):2212302. 10.1002/adma.202212302 36739173

[161] Nasseri R, Deutschman CP, Han L, Pope MA, Tam KC. Cellulose nanocrystals in smart and stimuli-responsive materials: A review. Mater Today Adv. 2020;5: Article 100055.

[162] Li Z, Lin Z. Recent advances in polysaccharide-based hydrogels for synthesis and applications. Aggregate. 2021;2(2): Article e21.

[163] Sood A, Dev A, Das SS, Kim HJ, Kumar A, Thakur VK, Han SS. Curcumin-loaded alginate hydrogels for cancer therapy and wound healing applications: A review. Int J Biol Macromol. 2023;232: Article 123283. 36657541 10.1016/j.ijbiomac.2023.123283

[164] Zhu J, Zheng S, Liu H, Wang Y, Jiao Z, Nie Y, Wang H, Liu T, Song K. Evaluation of anti-tumor effects of crocin on a novel 3D tissue-engineered tumor model based on sodium alginate/gelatin microbead. Int J Biol Macromol. 2021;174:339–351. 33529625 10.1016/j.ijbiomac.2021.01.181

[165] Olate-Moya F, Arens L, Wilhelm M, Mateos-Timoneda MA, Engel E, Palza H. Chondroinductive alginate-based hydrogels having graphene oxide for 3D printed scaffold fabrication. ACS Appl Mater Interfaces. 2020;12(4):4343–4357. 31909967 10.1021/acsami.9b22062

[166] Choi H, Kim Y, Kim S, Jung H, Lee S, Kim K, Han HS, Kim JY, Shin M, Son D. Adhesive bioelectronics for sutureless epicardial interfacing. Nat Electron. 2023;6:779–789.

[167] Kort-Mascort J, Bao G, Elkashty O, Flores-Torres S, Munguia-Lopez JG, Jiang T, Ehrlicher AJ, Mongeau L, Tran SD, Kinsella JM. Decellularized extracellular matrix composite hydrogel bioinks for the development of 3D bioprinted head and neck in vitro tumor models. ACS Biomater Sci Eng. 2021;7(11):5288–5300. 34661396 10.1021/acsbiomaterials.1c00812

[168] Louis F, Piantino M, Liu H, Kang DH, Sowa Y, Kitano S, Matsusaki M. Bioprinted vascularized mature adipose tissue with collagen microfibers for soft tissue regeneration. Cyborg Bionic Syst. 2021;2021:1412542. 36285131 10.34133/2021/1412542 PMC9494725

[169] Yi S, Liu Q, Luo Z, He JJ, Ma HL, Li W, Wang D, Zhou C, Garciamendez CE, Hou L, et al. Micropore-forming gelatin methacryloyl (gelma) bioink toolbox 2.0: Designable tunability and adaptability for 3d bioprinting applications. Small. 2022;18(25):2106357. 10.1002/smll.202106357 35607752

[170] Zhang Z, Yang J, Wang H, Wang C, Gu Y, Xu Y, Lee S, Yokota T, Haick H, Someya T, et al. A 10-micrometer-thick nanomeshreinforced permeable hydrogel skin sensor for long-term electrophysiological monitoring. Sci Adv. 2024;10(2):eadj5389. 38198560 10.1126/sciadv.adj5389 PMC10781413

[171] Balakrishnan G, Bhat A, Naik D, Kim JS, Marukyan S, Gido L, Ritter M, Khair AS, Bettinger CJ. Gelatin-based ingestible impedance sensor to evaluate gastrointestinal epithelial barriers. Adv Mater. 2023;35(17):e2211581. 36799712 10.1002/adma.202211581 PMC10192083

[172] Youn YH, Pradhan S, Kwon IK, Kundu SC, Reis RL, Yadavalli VK, Correlo VM. Micropatterned silk-fibroin/ eumelanin composite films for bioelectronic applications. ACS Biomater Sci Eng. 2021;7(6):2466–2474. 33851822 10.1021/acsbiomaterials.1c00216

[173] Ding J, Chen Z, Liu X, Tian Y, Jiang J, Qiao Z, Zhang Y, Xiao Z, Wei D, Sun J, et al. A mechanically adaptive hydrogel neural interface based on silk fibroin for high-efficiency neural activity recording. Mater Horiz. 2022;9(8):2215–2225. 35723211 10.1039/d2mh00533f

[174] Jiao Y, Zhang Y, Feng H, Li H, Wang Z, Wang P, Wang Y, Zheng N, Xie T, Ma Y, et al. A multifunctional bioelectronic device with switchable rigidity and reconfigurable shapes for comprehensive diagnosis. Adv Electron Mater. 2023;9(7):2201343.

[175] Shen K, Chen O, Edmunds JL, Piech DK, Maharbiz MM. Translational opportunities and challenges of invasive electrodes for neural interfaces. Nat Biomed Eng. 2023;7(4):424–442. 37081142 10.1038/s41551-023-01021-5

[176] Madhvapathy SR, Wang JJ, Wang H, Patel M, Chang A, Zheng X, Huang Y, Zhang ZJ, Gallon L, Rogers JA. Implantable bioelectronic systems for early detection of kidney transplant rejection. Science. 2023;381(6662):1105–1112. 37676965 10.1126/science.adh7726

[177] Veletić M, Apu EH, Simić M, Bergsland J, Balasingham I, Contag CH, Ashammakhi N. Implants with sensing capabilities. Chem Rev. 2022;122(21):16329–16363. 35981266 10.1021/acs.chemrev.2c00005

[178] Liang Q, Xia X, Sun X, Yu D, Huang X, Han G, Mugo SM, Chen W, Zhang Q. Highly stretchable hydrogels as wearable and implantable sensors for recording physiological and brain neural signals. Adv Sci. 2022;9(16):2201059. 10.1002/advs.202201059 PMC9165511 35362243

[179] Wang X, Ivanov AP, Edel JB. Biocompatible biphasic iontronics enable neuron-like ionic signal transmission. Research. 2024;7:0294. 38292443 10.34133/research.0294 PMC10826849

[180] Buzsáki G, Anastassiou CA, Koch C. The origin of extracellular fields and currents—EEG, ECoG, LFP and spikes. Nat Rev Neurosci. 2016;13(6):407–420. 10.1038/nrn3241 PMC4907333 22595786

[181] Wang Y, Yang X, Zhang X, Wang Y, Pei W. Implantable intracortical microelectrodes: Reviewing the present with a focus on the future. Microsyst Nanoeng. 2023;9:7. 36620394 10.1038/s41378-022-00451-6 PMC9814492

[182] Xie F, Xi Y, Xu Q, Liu J. Utah neural electrode technology for brain-computer interface. Acta Phys Chim Sin. 2020;36(12):2003014.

[183] Wu N, Wan S, Su S, Huang H, Dou G, Sun L. Electrode materials for brain–machine interface: A review. InfoMat. 2021;3(11):1174–1194.

[184] Hu Z, Niu Q, Hsiao BS, Yao X, Zhang Y. Bioactive polymer-enabled conformal neural interface and its application strategies. Mater Horiz. 2022;10(3):808–828. 10.1039/d2mh01125e 36597872

[185] Viana D, Walston ST, Masvidal-Codina E, Illa X, Rodríguez-Meana B, del Valle J, Hayward A, Dodd A, Loret T, Prats-Alfonso E, et al. Nanoporous graphene-based thin-film microelectrodes for in vivo high-resolution neural recording and stimulation. Nat Nanotechnol. 2024;19(4):514–523. 38212522 10.1038/s41565-023-01570-5 PMC11026161

[186] Liu S, Wang Y, Zhao Y, Liu L, Sun S, Zhang S, Liu H, Liu S, Li Y, Yang F, et al. A nanozyme-based electrode for high-performance neural recording. Adv Mater. 2023;36(6):e2304297. 37882151 10.1002/adma.202304297

[187] Li Y, Li N, Liu W, Prominski A, Kang S, Dai Y, Liu Y, Hu H, Wai S, Dai S, et al. Achieving tissue-level softness on stretchable electronics through a generalizable soft interlayer design. Nat Commun. 2023;14:4488. 37495580 10.1038/s41467-023-40191-3 PMC10372055

[188] Lu Y, Yang G, Wang S, Zhang Y, Jian Y, He L, Yu T, Luo H, Kong D, Xianyu Y, et al. Stretchable graphene–hydrogel interfaces for wearable and implantable bioelectronics. Nat Electron. 2024;7:51–65.

[189] Xia X, Liang Q, Sun X, Yu D, Huang X, Mugo SM, Chen W, Wang D, Zhang Q. Intrinsically electron conductive, antibacterial, and anti-swelling hydrogels as implantable sensors for bioelectronics. Adv Funct Mater. 2022;32(48):2208024.

[190] Vu PP, Vaskov AK, Lee C, Jillala RR, Wallace DM, Davis AJ, Kung TA, Kemp SWP, Gates DH, Chestek CA, et al. Long-term upper-extremity prosthetic control using regenerative peripheral nerve interfaces and implanted EMG electrodes. J Neural Eng. 2023;20: Article 026039. 10.1088/1741-2552/accb0c PMC10126717 37023743

[191] Muceli S, Poppendieck W, Holobar A, Gandevia S, Liebetanz D, Farina D. Blind identification of the spinal cord output in humans with high-density electrode arrays implanted in muscles. Sci Adv. 2022;8(46):eabo5040. 36383647 10.1126/sciadv.abo5040 PMC9668292

[192] Rossetti N, Hagler J, Kateb P, Cicoira F. Neural and electromyography PEDOT electrodes for invasive stimulation and recording. J Mater Chem C. 2021;9(23):7243–7263.

[193] Boys AJ, Carnicer-lombarte A, Güemes-Gonzalez A, van Niekerk DC, Hilton S, Barone DG, Proctor CM, Owens RM, Malliaras GG. 3D bioelectronics with a remodellable matrix for long-term tissue integration and recording. Adv Mater. 2023;35:2207847. 36458737 10.1002/adma.202207847 PMC11475589

[194] Zhang Z, Zhu Z, Zhou P, Zou Y, Yang J, Haick H, Wang Y. Soft bioelectronics for therapeutics. ACS Nano. 2023;17(18):17634–17667. 37677154 10.1021/acsnano.3c02513

[195] Jonsson A, Song Z, Nilsson D, Meyerson BA, Simon DT, Linderoth B, Berggren M. Therapy using implanted organic bioelectronics. Sci Adv. 2015;1(4): Article e1500039. 26601181 10.1126/sciadv.1500039 PMC4640645

[196] Huang S, He M, Yao C, Huang X, Ma D, Huang Q, Yang J, Liu F, Wen X, Wang J, et al. Petromyzontidae-biomimetic multimodal microneedles-integrated bioelectronic catheters for theranostic endoscopic surgery. Adv Funct Mater. 2023;33(15):2214485.

[197] Huang Y, Li H, Hu T, Li J, Yiu CK, Zhou J, Li J, Huang X, Yao K, Qiu X, et al. Implantable electronic medicine enabled by bioresorbable microneedles for wireless electrotherapy and drug delivery. Nano Lett. 2022;22(14):5944–5953. 35816764 10.1021/acs.nanolett.2c01997

[198] Lee J, Cho HR, Cha GD, Seo H, Lee S, Park CK, Kim JK, Qiao S, Wang L, Kang D, et al. Flexible, sticky, and biodegradable wireless device for drug delivery to brain tumors. Nat Commun. 2019;10(1):5205. 31729383 10.1038/s41467-019-13198-y PMC6858362

[199] Poinard B, Neo SZY, Yeo ELL, Heng HPS, Neoh KG, Kah JCY. Polydopamine nanoparticles enhance drug release for combined photodynamic and photothermal therapy. ACS Appl Mater Interfaces. 2018;10(25):21125–21136. 29871485 10.1021/acsami.8b04799

[200] Zhang T, Liang H, Wang Z, Qiu C, Peng YB, Zhu X, Li J, Ge X, Xu J, Huang X, et al. Piezoelectric ultrasound energy–harvesting device for deep brain stimulation and analgesia applications. Sci Adv. 2022;8:eabk0159. 35427156 10.1126/sciadv.abk0159 PMC9012468

[201] Lee H, Lee Y, Song C, Cho HR, Ghaffari R, Choi TK, Kim KH, Lee YB, Ling D, Lee H, et al. An endoscope with integrated transparent bioelectronics and theranostic nanoparticles for colon cancer treatment. Nat Commun. 2015;6:10059. 26616435 10.1038/ncomms10059 PMC4674684

[202] Guan S, Tian H, Yang Y, Liu M, Ding J, Wang J, Fang Y. Self-assembled ultraflexible probes for long-term neural recordings and neuromodulation. Nat Protoc. 2023;18(6):1712–1744. 37248393 10.1038/s41596-023-00824-9

[203] Zou L, Tian H, Guan S, Ding J, Gao L, Wang J, Fang Y. Self-assembled multifunctional neural probes for precise integration of optogenetics and electrophysiology. Nat Commun. 2021;12(1):5871. 34620851 10.1038/s41467-021-26168-0 PMC8497603

[204] Huang W, Chi H-S, Lee Y-C, Lo Y-C, Liu T-C, Chiang M-Y, Chen H-Y, Li S-J, Chen Y-Y, Chen S-Y. Gene-embedded nanostructural biotic-abiotic optoelectrode arrays applied for synchronous brain optogenetics and neural signal recording. ACS Appl Mater Interfaces. 2019;11:11270–11282. 30844235 10.1021/acsami.9b03264

[205] Cho M, Han J, Suh J, Kim JJ, Ryu JR, Min IS, Sang M, Lim S, Kim TS, Kim K, et al. Fully bioresorbable hybrid opto-electronic neural implant system for simultaneous electrophysiological recording and optogenetic stimulation. Nat Commun. 2024;15:2000. 10.1038/s41467-024-45803-0 PMC10917781 38448437

[206] Ferrigno B, Bordett R, Duraisamy N, Moskow J, Arul MR, Rudraiah S, Nukavarapu SP, Vella AT, Kumbar SG. Bioactive polymeric materials and electrical stimulation strategies for musculoskeletal tissue repair and regeneration. Bioact Mater. 2020;5(3):468–485. 32280836 10.1016/j.bioactmat.2020.03.010 PMC7139146

[207] Lei H, Fan D. Conductive, adaptive, multifunctional hydrogel combined with electrical stimulation for deep wound repair. Chem Eng J. 2021;421(Part 1): Article 129578.

[208] Woods GA, Rommelfanger NJ, Hong G. Review bioinspired materials for in vivo bioelectronic neural interfaces. Matter. 2020;3(4):1087–1113. 33103115 10.1016/j.matt.2020.08.002 PMC7583599

[209] Wang W, Jiang Y, Zhong D, Zhang Z, Choudhury S, Lai J-C, Gong H, Niu S, Yan X, Zheng Y, et al. Neuromorphic sensorimotor loop embodied by monolithically integrated, low-voltage, soft e-skin. Science. 2023;742:735–742. 10.1126/science.ade0086 37200416

[210] Dong Y, Wang S, Huang Q, Berg RW, Li G, He J. Neural decoding for intracortical brain-computer interfaces. Cyborg Bionic Syst. 2023;4:0044. 37519930 10.34133/cbsystems.0044 PMC10380541

[211] Tai P, Ding P, Wang F, Gong A, Li T, Zhao L, Su L, Fu Y. Brain-computer interface paradigms and neural coding. Front Neurosci. 2023;17:1345961. 38287988 10.3389/fnins.2023.1345961 PMC10822902

[212] Leber M, Körner J, Reiche CF, Yin M, Bhandari R, Franklin R, Negi S, Solzbacher F. Advances in penetrating multichannel microelectrodes based on the Utah array platform. Adv Exp Med Biol. 2019;1101:1–40. 31729670 10.1007/978-981-13-2050-7_1

[213] Liu D, Xu X, Li D, Li J, Yu X, Ling Z, Hong B. Intracranial brain-computer interface spelling using localized visual motion response. NeuroImage. 2022;258: Article 119363. 35688315 10.1016/j.neuroimage.2022.119363

[214] Han JK, Yun SY, Lee SW, Yu JM, Choi YK. A review of artificial spiking neuron devices for neural processing and sensing. Adv Funct Mater. 2022;32(33):2204102.

[215] Zhang M, Tang Z, Liu X, Van der Spiegel J. Electronic neural interfaces. Nat Electron. 2020;3(4):191–200.

[216] Liu X, Qiu F, Hou L, Wang X. Review of noninvasive or minimally invasive deep brain stimulation. Front Behav Neurosci. 2022;15: Article 820017. 35145384 10.3389/fnbeh.2021.820017 PMC8823253

[217] Li Y, Jiang Y, Lan L, Ge X, Cheng R, Zhan Y, Chen G, Shi L, Wang R, Zheng N, et al. Optically-generated focused ultrasound for noninvasive brain stimulation with ultrahigh precision. Light Sci Appl. 2022;11(1):321. 36323662 10.1038/s41377-022-01004-2 PMC9630534

[218] Ji B, Ge C, Guo Z, Wang L, Wang M, Xie Z, Xu Y, Li H, Yang B, Wang X, et al. Flexible and stretchable opto-electric neural interface for low-noise electrocorticogram recordings and neuromodulation in vivo. Biosens Bioelectron. 2020;153: Article 112009. 31989934 10.1016/j.bios.2020.112009

[219] Wu X, Jiang Y, Rommelfanger NJ, Yang F, Zhou Q, Yin R, Liu J, Cai S, Ren W, Shin A, et al. Tether-free photothermal deep-brain stimulation in freely behaving mice via wide-field illumination in the near-infrared-II window. Nat Biomed Eng. 2022;6(6):754–770. 35314800 10.1038/s41551-022-00862-w PMC9232843

[220] Missey F, Donahue MJ, Weber P, Ngom I, Acerbo E, Botzanowski B, Migliaccio L, Jirsa V, Głowacki ED, Williamson A. Laser-driven wireless deep brain stimulation using temporal interference and organic electrolytic photocapacitors. Adv Funct Mater. 2022;32:2200691.

[221] Cafarelli A, Marino A, Vannozzi L, Puigmartí-Luis J, Pané S, Ciofani G, Ricotti L. Piezoelectric nanomaterials activated by ultrasound: The pathway from discovery to future clinical adoption. ACS Nano. 2021;15(7):11066–11086. 34251189 10.1021/acsnano.1c03087 PMC8397402

[222] Chen P, Wang Q, Wan X, Yang M, Liu C, Xu C, Hu B, Feng J, Luo Z. Wireless electrical stimulation of the vagus nerves by ultrasound-responsive programmable hydrogel nanogenerators for anti-inflammatory therapy in sepsis. Nano Energy. 2021;89(Part A): Article 106327.

[223] Nanogenerators P, Chen P, Cheng C, Yang X, Sha T-T, Zou X, Zhang F, Jiang W, Xu Y, Cao X, et al. Wireless deep brain stimulation by ultrasound-responsive molecular. ACS Nano. 2023;17:25625–25637. 38096441 10.1021/acsnano.3c10227

[224] Townsend N, Kazakiewicz D, Lucy Wright F, Timmis A, Huculeci R, Torbica A, Gale CP, Achenbach S, Weidinger F, Vardas P. Epidemiology of cardiovascular disease in Europe. Nat Rev Cardiol. 2022;19(2):133–143. 34497402 10.1038/s41569-021-00607-3

[225] Parati G, Torlasco C, Pengo M, Bilo G, Ochoa JE. Blood pressure variability: Its relevance for cardiovascular homeostasis and cardiovascular diseases. Hypertens Res. 2020;43(7):609–620. 32203448 10.1038/s41440-020-0421-5

[226] Kawamura Y, Yokoyama H, Kitayama K, Miura N, Hamadate M, Nagawa D, Nozaka M, Nakata M, Nishizaki F, Hanada K, et al. Clinical impact of complete atrioventricular block in patients with ST-segment elevation myocardial infarction. Clin Cardiol. 2021;44(1):91–99. 33179796 10.1002/clc.23510 PMC7803372

[227] Li X, Zhang Y, Ren X, Wang Y, Chen D, Li Q, Huo M, Shi J. Ischemic microenvironment-responsive therapeutics for cardiovascular diseases. Adv Mater. 2021;33(52):202105348. 10.1002/adma.202105348 34623714

[228] Wang P, Li J, Zhang W, Ren Y, Ma J, Li S, Tan X, Chi B. 3D printed heart valve mediated nitric oxide sustained release reduced potential for calcification and inflammatory capacity. Chem Eng J. 2023;469: Article 143892.

[229] Zhao T, Wu W, Sui L, Huang Q, Nan Y, Liu J, Ai K. Reactive oxygen species-based nanomaterials for the treatment of myocardial ischemia reperfusion injuries. Bioact Mater. 2022;7:47–72. 34466716 10.1016/j.bioactmat.2021.06.006 PMC8377441

[230] Choi H, Kim Y, Kim S, Jung H, Lee S, Kim K, Han H-S, Kim JY, Shin M, Son D. Adhesive bioelectronics for sutureless epicardial interfacing. Nat Electron. 2023;6(10):779–789.

[231] Sunwoo SH, Han SI, Jung D, Kim M, Nam S, Lee H, Choi S, Kang H, Cho YS, Yeom DH, et al. Stretchable low-impedance conductor with Ag-Au-Pt core-shell-shell nanowires and in situ formed Pt nanoparticles for wearable and implantable device. ACS Nano. 2023;17(8):7550–7561. 37039606 10.1021/acsnano.2c12659

[232] Wang C, Shi Q, Lee C. Advanced implantable biomedical devices enabled by triboelectric nanogenerators. Nano. 2022;12(8):1366. 10.3390/nano12081366 PMC9032723 35458075

[233] Jin P, Fu J, Wang F, Zhang Y, Wang P, Liu X, Jiao Y, Li H, Chen Y, Ma Y, et al. A flexible, stretchable system for simultaneous acoustic energy transfer and communication. Sci Adv. 2021;7(40):abg2507. 10.1126/sciadv.abg2507 PMC8480923 34586839

[234] Ryu H, Wang X, Xie Z, Kim J, Liu Y, Bai W, Song Z, Song JW, Zhao Z, Kim J, et al. Materials and design approaches for a fully bioresorbable, electrically conductive and mechanically compliant cardiac patch technology. Adv Sci. 2023;10(27):2303429. 10.1002/advs.202303429 PMC10520666 37518771

[235] Xie X, Xu Z, Yu X, Jiang H, Li H, Feng W. Liquid-in-liquid printing of 3D and mechanically tunable conductive hydrogels. Nat Commun. 2023;14:4289. 37463898 10.1038/s41467-023-40004-7 PMC10354067

[236] Kalidasan V, Yang X, Xiong Z, Li RR, Yao H, Godaba H, Obuobi S, Singh P, Guan X, Tian X, et al. Wirelessly operated bioelectronic sutures for the monitoring of deep surgical wounds. Nat Biomed Eng. 2021;5(10):1217–1227. 34654900 10.1038/s41551-021-00802-0

[237] Xue F, Zhao S, Tian H, Qin H, Li X, Jian Z, du J, Li Y, Wang Y, Lin L, et al. Two way workable microchanneled hydrogel suture to diagnose, treat and monitor the infarcted heart. Nat Commun. 2024;15:864. 38286997 10.1038/s41467-024-45144-y PMC10824767

[238] Wang X, Zhao H, Liu Z, Wang Y, Lin D, Chen L, Dai J, Lin K, Shen SG. Polydopamine nanoparticles as dual-task platform for osteoarthritis therapy: A scavenger for reactive oxygen species and regulator for cellular powerhouses. Chem Eng J. 2021;417: Article 129284.

[239] Wang T, Ouyang H, Luo Y, Xue J, Wang E, Zhang L, Zhou Z, Liu Z, Li X, Tan S, et al. Rehabilitation exercise–driven symbiotic electrical stimulation system accelerating bone regeneration. Sci Adv. 2024;10:eadi6799. 38181077 10.1126/sciadv.adi6799 PMC10776020

[240] Hu ZC, Lu JQ, Zhang TW, Liang HF, Yuan H, Su DH, Ding W, Lian RX, Ge YX, Liang B, et al. Piezoresistive MXene/silk fibroin nanocomposite hydrogel for accelerating bone regeneration by reestablishing electrical microenvironment. Bioact Mater. 2023;22:1–17. 36203961 10.1016/j.bioactmat.2022.08.025 PMC9513113

[241] Shanker A, Bashashati M, Rezaie A. Gastric electrical stimulation for treatment of refractory gastroparesis: The current approach to management. Curr Gastroenterol Rep. 2021;23(2):2. 33483775 10.1007/s11894-020-00803-0 PMC7822763

[242] Ramadi KB, McRae JC, Selsing G, Su A, Fernandes R, Hickling M, Rios B, Babaee S, Min S, Gwynne D, et al. Bioinspired, ingestible electroceutical capsules for hunger-regulating hormone modulation. Sci Robot. 2023;8(77):eade9676. 37099636 10.1126/scirobotics.ade9676 PMC10508349

[243] Cheng S, Hang C, Ding L, Jia L, Tang L, Mou L, Qi J, Dong R, Zheng W, Zhang Y, et al. Article electronic blood vessel. Matter. 2020;3(5):1664–1684.

[244] Wei S, Jiang A, Sun H, Zhu J, Jia S, Liu X, Xu Z, Zhang J, Shang Y, Fu X, et al. Shape-changing electrode array for minimally invasive large-scale intracranial brain activity mapping. Nat Commun. 2024;15(1):715. 38267440 10.1038/s41467-024-44805-2 PMC10808108

[245] Dai J, Wang B, Chang Z, Lu X, Nie J, Ren Q, Lv Y, Rotenberg MY, Fang Y. Injectable mesh-like conductive hydrogel patch for elimination of atrial fibrillation. Adv Healthc Mater. 2024;13(17):e2303219. 38198617 10.1002/adhm.202303219

[246] Ju J, Hu N, Cairns DM, Liu H, Timko BP. Photo-cross-linkable, insulating silk fibroin for bioelectronics with enhanced cell affinity. Proc Natl Acad Sci USA. 2020;117(27):15482–15489. 32571918 10.1073/pnas.2003696117 PMC7376572

